# Molar Mass Improves the Performance of n‑Type Organic Electrochemical Transistors

**DOI:** 10.1021/acs.chemmater.5c00949

**Published:** 2025-07-07

**Authors:** Dominik Stegerer, Tiefeng Liu, Miao Xiong, Han-Yan Wu, Min Gyu Kang, Han Young Woo, Simone Fabiano, Michael Sommer

**Affiliations:** † Institut für Chemie, 38869 Technische Universität Chemnitz, 09111 Chemnitz, Germany; ‡ Laboratory of Organic Electronics, Department of Science and Technology, 4566 Linköping University, SE-601 74 Norrköping, Sweden; § Wallenberg Initiative Materials Science for Sustainability, Department of Science and Technology, Linköping University, SE-601 74 Norrköping, Sweden; ∥ Department of Chemistry, College of Science, 34973 Korea University, 136-713 Seoul, Republic of Korea; ⊥ Center for Materials, Architectures and Integration of Nanomembranes (MAIN), Technische Universität Chemnitz, 09126 Chemnitz, Germany

## Abstract

We report on the synthesis and use of two side-chain-free ionenes with varying heteroatoms, PFu and PTh, for n-type accumulation-mode OECTs. Compared to PTh, PFu is more challenging to make, less stable, and shows increased water solubility. The optical properties and surface morphologies of the two derivatives are comparable, but their microstructures vary distinctly in terms of ordering and backbone orientation. While the backbones of PTh show a preferential face-on orientation, PFu is significantly less ordered. The OECT performance of PTh is improved by 1 order of magnitude compared to PFu, as indicated by μC* values of 116.16 and 10.66 F cm^–1^ V^–1^ s^–1^, respectively. Further increasing the molar mass of PTh doubles the performance, resulting in a record-high μC* value of 225.71 F cm^–1^ V^–1^ s^–1^ and a high μ value of 0.58 cm^2^ V^–1^ s^–1^, highlighting the crucial role of molecular weight control for enhancing device performance.

## Graphical abstract

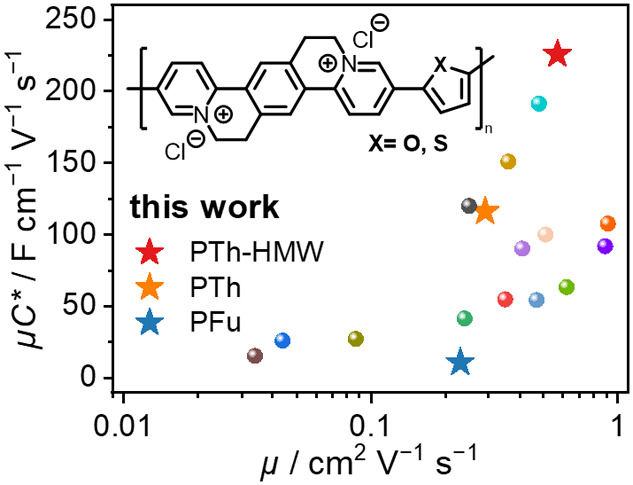

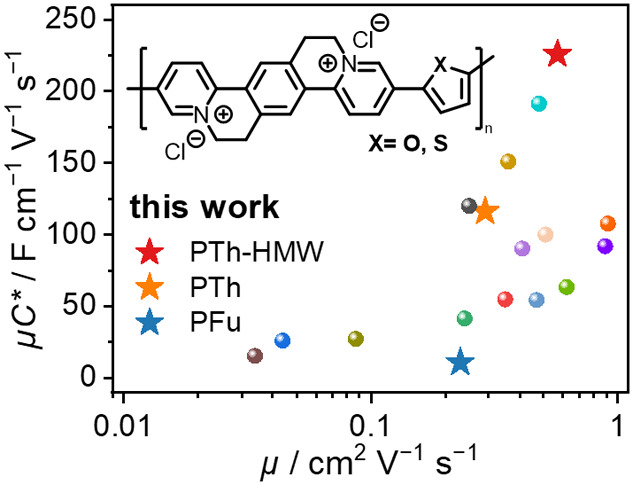

## 1 Introduction

Organic electrochemical transistors (OECTs) have garnered widespread attention due to their low working bias, low power consumption, high sensitivity, biocompatibility, and mixed ionic-electronic response.^1,2^ These characteristics make OECTs highly promising for applications in chemical and biological sensing as well as neuromorphic computing.^3,4^ The operation of the OECTs is based on electrochemical doping/dedoping processes of the active material in the channel, where the ions from an electrolyte penetrate into the bulk of the material, thus modulating its conductance in response to the bias of the gate voltage. Consequently, the figure of merit μC*, which represents the product of charge-carrier mobility (μ) and ion-uptake capability (C*), has been established as a key benchmark for evaluating channel materials.^5,6^

To fabricate high-performance and power-efficient OECT-based circuits or sensors, it is crucial to develop both p- and n-type OECT materials with high μC*. To date, p-type channel materials have made great progress with excellent μC* values exceeding 500 F cm^–1^ V^–1^ s^–1^.^7,8^ In contrast, n-type OECT materials have lagged far behind, with only a few polymers achieving μC* values over 100 F cm^–1^ V^–1^ s^–1^. Table S1 in the Supporting Information provides an overview of the literature. Most efforts have been devoted to designing n-type OECT materials by introducing hydrophilic oligo­(ethylene glycol) (OEG) side chains into electron-deficient fused aromatic building blocks.^9–11^ On the one hand, this strategy allows ions to penetrate into the channel material, enabling ion transport and volumetric charging of the bulk of the material. On the other hand, excessive water uptake can, in some cases, compromise device stability and reduce electronic mobility and therefore must be carefully controlled. One possibility to tailor the hydrophilicity of the material is to use mixed OEG and aliphatic side chains.^12,13^ Side-chain-free polymers have emerged as promising alternatives for n-type OECTs. The absence of side chains enables these materials to accommodate a higher ion density during electrochemical reduction,^14,15^ but a modulation of solubility and hydrophilicity would have to be accomplished by the choice of the monomers or chain length. One of the earliest breakthroughs in this category was poly­(benzimidazobenzophenanthroline) (BBL), which exhibited a record-high C* among n-type OECT materials.^16,17^ Moreover, it was demonstrated that increasing the molecular weight by 1 order of magnitude resulted in a significant enhancement in μ and μC*, while C* itself remained largely unaffected.^17^ Such an improvement in electron mobility with molecular weight is also seen in field-effect transistor studies, in which the electron mobility can be increased by more than 1 order of magnitude upon optimizing chain length.^18^ More recently, the cationic conjugated polymer P­(PyV)-H was developed as a hydrogel, achieving a high C* of 485 F cm^–3^ and a μ of 0.25 cm^2^ V^–1^ s^–1^, resulting in a μC* of 120 F cm^–1^ V^–1^ s^–1^.^19^ These excellent performances highlight the potential of side-chain-free materials in the design of high-performance n-type OECTs. The bispyridinium phenylene motif (PymPhPym) contained in P­(PyV)-H is known for its highly electron-deficient nature, resulting in a strongly negative LUMO energy level suitable for n-doping,^20,21^ but has not been widely explored in combination with other comonomers and for use in OECTs. We therefore anticipated that further modulation of PymPhPym-based copolymers in terms of comonomer and chain length is beneficial for improving the performance of OECT devices.

Here we report on the synthesis and use of two n-type copolymers termed PFu and PTh for high-performance OECTs. The copolymers contain the PymPhPym motif in combination with furan (Fu) and thiophene (Th) (Scheme 1). The furan unit is expected to lead to enhanced coplanarity due to reduced steric hindrance arising from its smaller size, but it is also less aromatic and may cause a stronger localization of polarons.^22,23^ However, the synthesis of PFu poses more difficulties in obtaining high molecular weights, and the resulting material is less stable. While both polymers exhibit comparable optoelectronic properties, PTh displays a significantly better ordered thin-film microstructure with predominantly face-on-oriented chains. Both materials show n-type OECT characteristics; however, PTh largely outperforms PFu by nearly an order of magnitude, as indicated by their respective μC* values of 116.16 and 10.66 F cm^–1^ V^–1^ s^–1^, respectively. Furthermore, increasing the molecular weight of PTh leads to a doubling of the OECT performance, achieving a record-high μC* value of 225.71 F cm^–1^ V^–1^ s^–1^ and a high μ of 0.58 cm^2^ V^–1^ s^–1^. This underlines the still underrepresented role of chain length of conjugated polymers on electrical properties and performance.

**Scheme 1:**
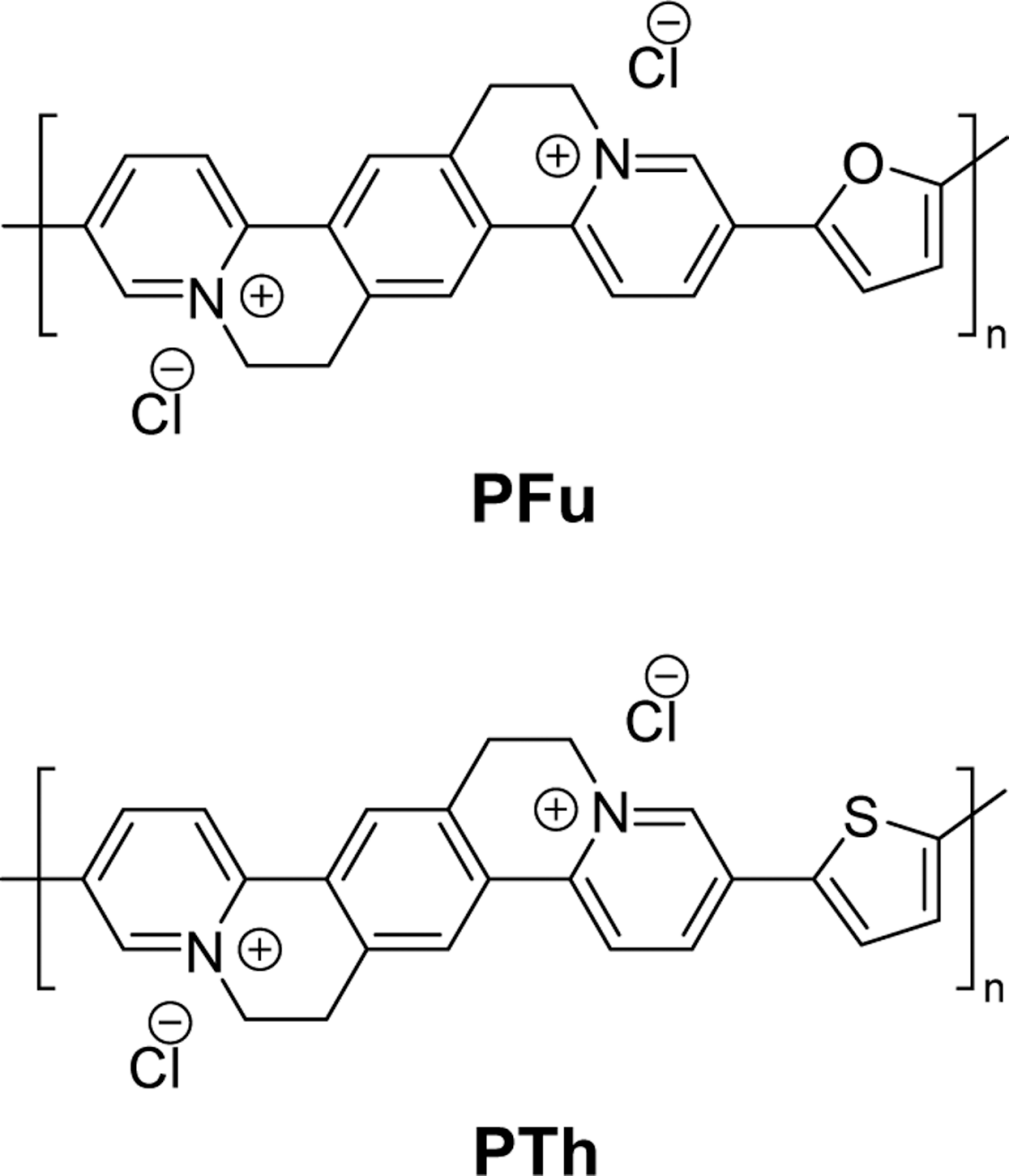
Chemical Structures of PTh and PFu

## 2 Experimental Section

All details of starting materials, monomer synthesis, polymerizations, methods, and characterization are described in the Supporting Information. This file also contains NMR spectra, size exclusion chromatography curves, additional absorption data, spectro-electrochemistry data, solubility tests, structural analysis, atomic force microscopy images, further OECT device data, and additional references.

## 3 Results and Discussion

The monomer PyPhPy was prepared using a modified protocol (Scheme S1).^20^ Notably, the modification of the reaction procedure resulted in an increased overall yield and high purity of the monomers, which is key for achieving high-molar-mass materials (for details, see the Supporting Information). The synthesis of the nonquaternized polymers P­(PyPhPyTh) and P­(PyPhPyFu) (Scheme S2) was adapted using reaction conditions previously optimized for other electron-deficient monomers.^24^ For P­(PyPhPyTh), the molar mass was modulated by adjusting the stoichiometry, yielding different number-average molar masses determined by size exclusion chromatography (SEC), *M*_n,SEC_, of up to ∼52 kg/mol (Table 1 and Figure S1a). The high-molecular-weight variant of P­(PyPhPyTh) is referred to as P­(PyPhPyTh)-HMW.

**Table 1:** Polymer Characterization Data

polymer (nonquaternized)	*M*_n_^a^/kg mol^–1^	*M*_w_^a^/kg mol^–1^	*D̵* ^a^	DP_n,NMR_ ^b^	*M*_n,NMR_^b^/kg mol^–1^	polymer (quaternized)	*M*_n,NMR_^c^/kg mol^–1^	*E*_red_^d^/V	LUMO^e^/eV	HOMO^f^/eV
P(PyPhPyFu)	12.4	25.1	2.0	19	12	PFu	8.3	–0.79	–4.01	–6.24
P(PyPhPyTh)	21.0	50.8	2.4	22	14	PTh	9.7	–0.70	–4.10	–6.38
P(PyPhPyTh)-HMW	52.2	117	2.2	n.d.	n.d.	PTh-HMW	n.d.	–0.70	–4.10	–6.36

a From SEC in THF.

b From ^1^H NMR end-group analysis.

c Recalculated from *M*_n,NMR_ values obtained from end-group analysis.

d Reduction onset potentials (*E*_onset_) of polymer films on ITO substrates from CV vs. Fc/Fc^+^.

e From CV using −4.80 eV for Fc/Fc^+^.

f From HOMO = LUMO − *E*_g,film,opt_ using 2.23 eV, 2.28 eV, and 2.26 eV for PFu, PTh, and PTh-HMW, respectively. N.d. = not determined.

As the *M*_n,SEC_ values of P­(PyPhPyTh) and P­(PyPhPyFu) differed by a factor of ∼ 1.7 (Table 1) despite efforts to maintain stoichiometry for both polymers, we analyzed end-group signals of the ^1^H NMR spectra of the polymers to extract absolute *M*_n,NMR_ values. Figure 1 shows a comparison of polymer and monomer spectra with full-range spectra provided in Figure S2. The chemical shifts of the Th and Fu protons differed significantly (Figure 1b,c), as also reflected in the ^1^H NMR spectra of the monomers (Figure S2a,b). The ^1^H NMR spectra of the quaternized monomer and polymers PFu and PTh are shown in Figure 1d–f, the latter two of which are characterized by broad signals caused by the rigid chain structure. Comparison of these broad spectra to the quaternized model compound PymPhPym (Figure 1d) also suggests the presence of end-groups, but interpretation or even quantification was not possible (Figure S3). A detailed analysis of the end-groups was therefore carried out using the spectra of the nonquaternized polymers. Figure 1g,h displays the aromatic region of P­(PyPhPyTh) and P­(PyPhPyFu), where end-group signals are dominated by PyPhPyBr chain ends. This was expected from the slight excess of the halide monomer used (see Supporting Information for details) and allowed us to determine absolute degrees of polymerization *DP*_n_ as well as *M*_n,NMR_ values for the samples P­(PyPhPyTh) and P­(PyPhPyFu). For P­(PyPhPyTh)-HMW, end-groups were not visible, and therefore, these values could not be determined (Table 1). Other potential end-group signals were negligibly small and therefore not considered for the calculation of *M*_n,NMR_, which therefore represents an upper limit. In contrast to the different *M*_n,SEC_ values, the absolute *M*_n,NMR_ values of P­(PyPhPyTh) and P­(PyPhPyFu) are 14 and 12 kg/mol, respectively, and thus are more similar. The smaller value of the *M*_n,SEC_ of P­(PyPhPyFu) is a combined effect of a lower molar mass and a possibly smaller persistence length in solution as a result of the geometry of furan, causing a stronger kinking of the backbone.^25^ Absolute *M*_n,NMR_ values for the quaternized polymers were obtained from the *M*_n,NMR_ values of the corresponding precursors; for this reason, a value for PTh-HMW could not be extracted (Table 1). However, by judging from the different molar masses of P­(PyPhPyTh) and P­(PyPhPyTh)-HMW, we estimated that the *M*_n,NMR_ of PTh-HMW is larger by a factor of ∼2–2.5 compared to PTh.

**Figure 1:**
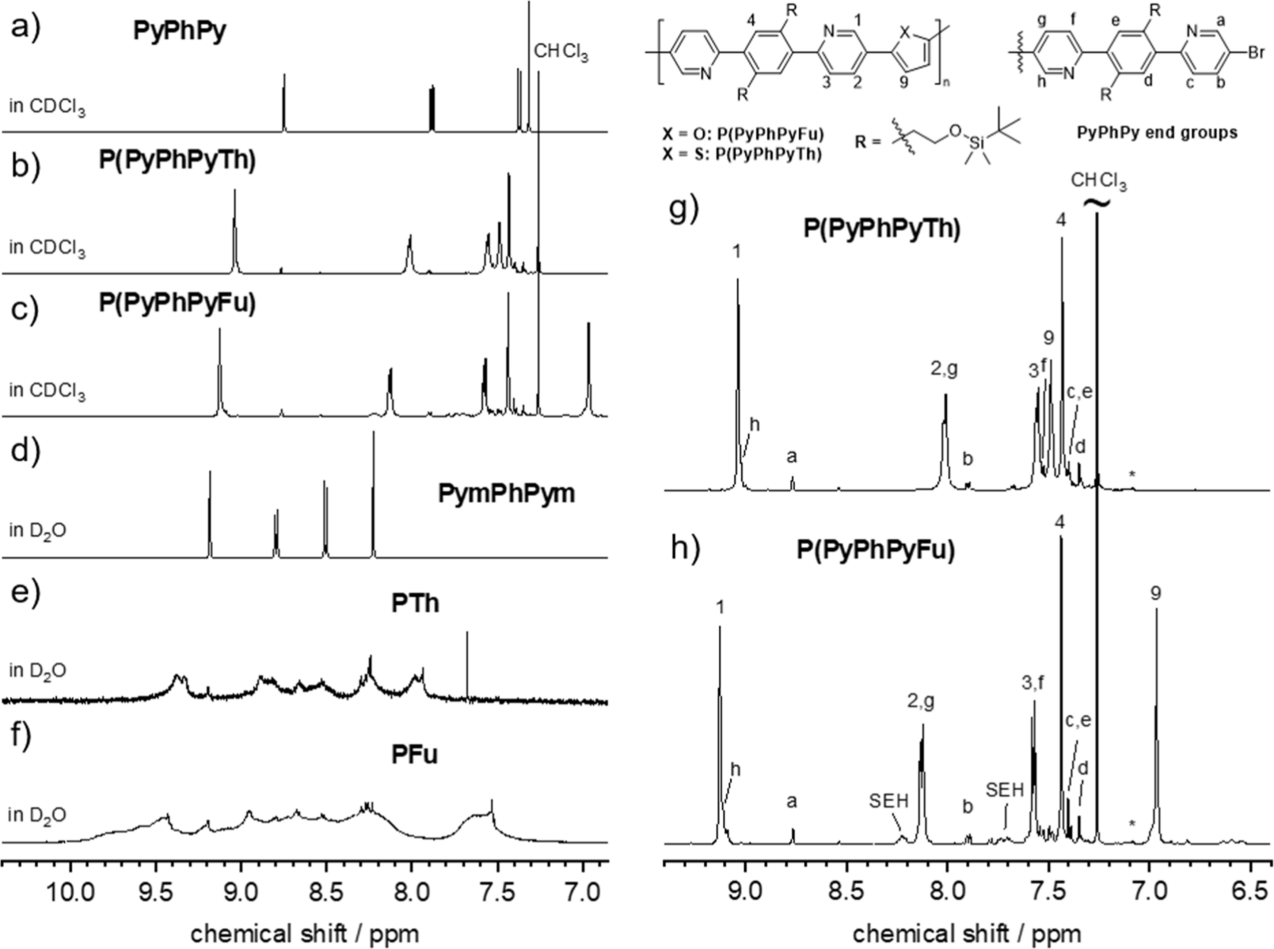
^1^H NMR spectra (selected regions) of the (a) monomer, (b,c) nonquaternized polymers, (d) quaternized monomer, and (e,f) quaternized polymers. (g,h) Enlarged regions with details of end-group analysis. SEH: silyl ether hydrolysis.

P­(PyPhPyTh), P­(PyPhPyTh)-HMW, and P­(PyPhPyFu) were quaternized using SOCl_2_ in a chloroform–acetonitrile mixture, following a modified procedure (for details see the Supporting Information and Scheme S2).^20^ Critically, CHCl_3_ was used as a cosolvent to enhance the solubility of the nonquaternized polymer in the mixture. All resulting quaternized polymers, PFu, PTh, and PTh-HMW, were soluble in 2,2,2-trifluoroethanol (TFE) but exhibited distinct differences in water solubility (Figure S4). While PFu demonstrated excellent water solubility, PTh had relatively low solubility in water, though still higher than in other common polar protic or aprotic solvents. The addition of electrolytes and the use of suitable cosolvents were found to improve the solubility of both quaternized polymers. PTh-HMW was completely insoluble in water. Because of the higher dipole moment of furan, PFu exhibited superior water solubility compared to PTh. These trends highlight that the nature of the comonomer as well as chain length are potent measures to control the interaction and penetration of the surrounding medium with/into the active layer of OECT devices.

Interestingly, neither the choice of comonomers nor molecular weight affected the optical absorption properties of the nonquaternized polymers in solution (Figure S1b). The UV–vis spectra of P­(PyPhPyTh), P­(PyPhPyTh)-HMW, and P­(PyPhPyFu) in THF solution exhibited nearly identical absorption profiles, with a maximum at 371 nm. The UV–vis spectra of PFu and PTh exhibited similar absorption characteristics both in solution (Figure 2a) and in thin film (Figure 2b). The absorption bands between 320 and 340 nm and between 400 and 550 nm arise from π–π* transitions of the PymPhPym motif and charge transfer bands from the alternating donor–acceptor backbone, respectively. This is also consistent with the absorption spectra of the nonquaternized precursor polymers, where a single band is present (cf. Figure S1b). PTh-HMW was not soluble in D_2_O. The thin film spectra were slightly red-shifted compared to the solution spectra and featured similar onsets of absorption. Upon comparing the spectra of PFu and PTh, a small bathochromic shift was seen for PTh both in solution and film, suggesting that thiophene is a stronger donor. Vibronic bands were visible in both solution and thin film spectra, with a notably stronger 0–0 vibronic band in films of PTh-HMW compared to PTh.

**Figure 2:**
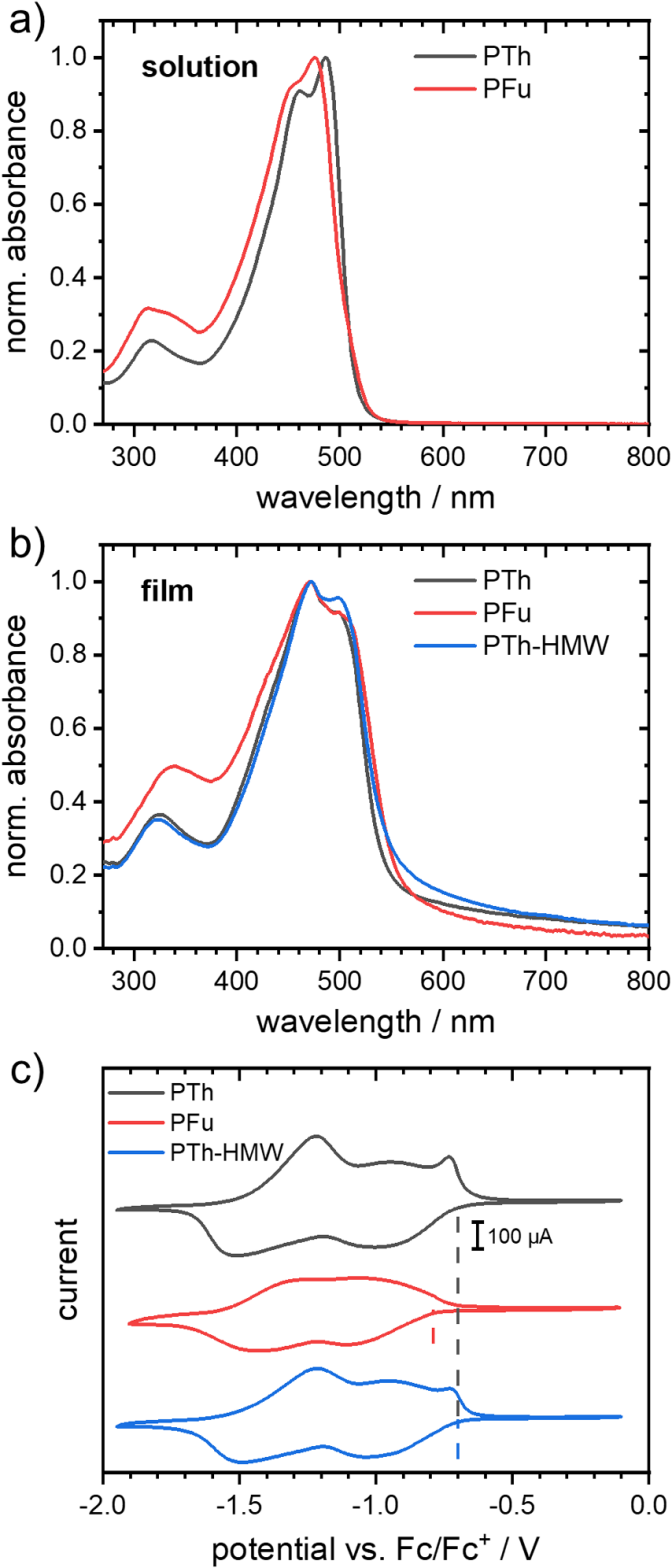
UV–vis spectra of the polymers in (a) D_2_O and (b) thin films. (c) Cyclic voltammograms of the reduction process of the polymers in thin films on ITO-coated substrates. The dashed lines indicate reduction potentials used to calculate the LUMO energy levels.

The electrochemical reduction potential of the polymers was investigated by using cyclic voltammetry (CV), with the results presented in Figure 2c and Table 1. The shape of the reduction curve of PTh is typical for a two-electron reduction, yielding a LUMO energy level of −4.10 eV.^20^ The LUMO energy level of PFu was slightly more positive at −4.01 eV, while the shape of its reduction curve closely resembled that of the two-electron reduction of PTh. The CV curve of PTh-HMW was identical to that of PTh. These results suggest that the low LUMO energy level primarily originates from the electron-deficient PymPhPym unit.^20,26^ Furthermore, the effect of the Th and Fu comonomer units appeared to have a minor impact on the optoelectronic properties of the polymers.

The spectro-electrochemical redox behavior was further investigated to map differences in the absorption of electrochemically reduced species (Figure S5). The inspection of the polaron bands of the different polymers revealed residual absorption of neutral PFu chains at −1.0 V. This is a direct result of the increased solubility of PFu and the resulting partial dissolution of the film, making the dissolved fraction unavailable for reduction. Also, the polaron absorption band of PFu evolved at somewhat more negative voltages compared with PTh, which we ascribed to the difference in the onsets of the two LUMO levels. Thus, at a given voltage, PFu is not in the same reduction state as the other two polymers. Furthermore, the polaron absorption of PTh extended much more into the NIR region compared to PFu. A possible reason is the likely more kinked backbone structure of PFu and the greater diene character of furan compared to thiophene, which can lower the conjugation length.

Grazing-incidence wide-angle X-ray scattering (GIWAXS) was employed to analyze the thin-film microstructures. As shown in Figure 3b,d,e, PTh predominantly adopted a face-on orientation, characterized by a (100) lamellar stacking peak located in-plane at around *q*_*xy*_ = 0.715 Å^–1^ (*d*-spacing = 8.788 Å) and a strong (010) π–π stacking peak oriented out-of-plane at *q*_*z*_ = 1.732 Å^–1^ (*d*-spacing = 3.628 Å). A minor content of the edge-on orientation was also visible (Figure 3e). These structural features are consistent with previously reported results.^27^

**Figure 3:**
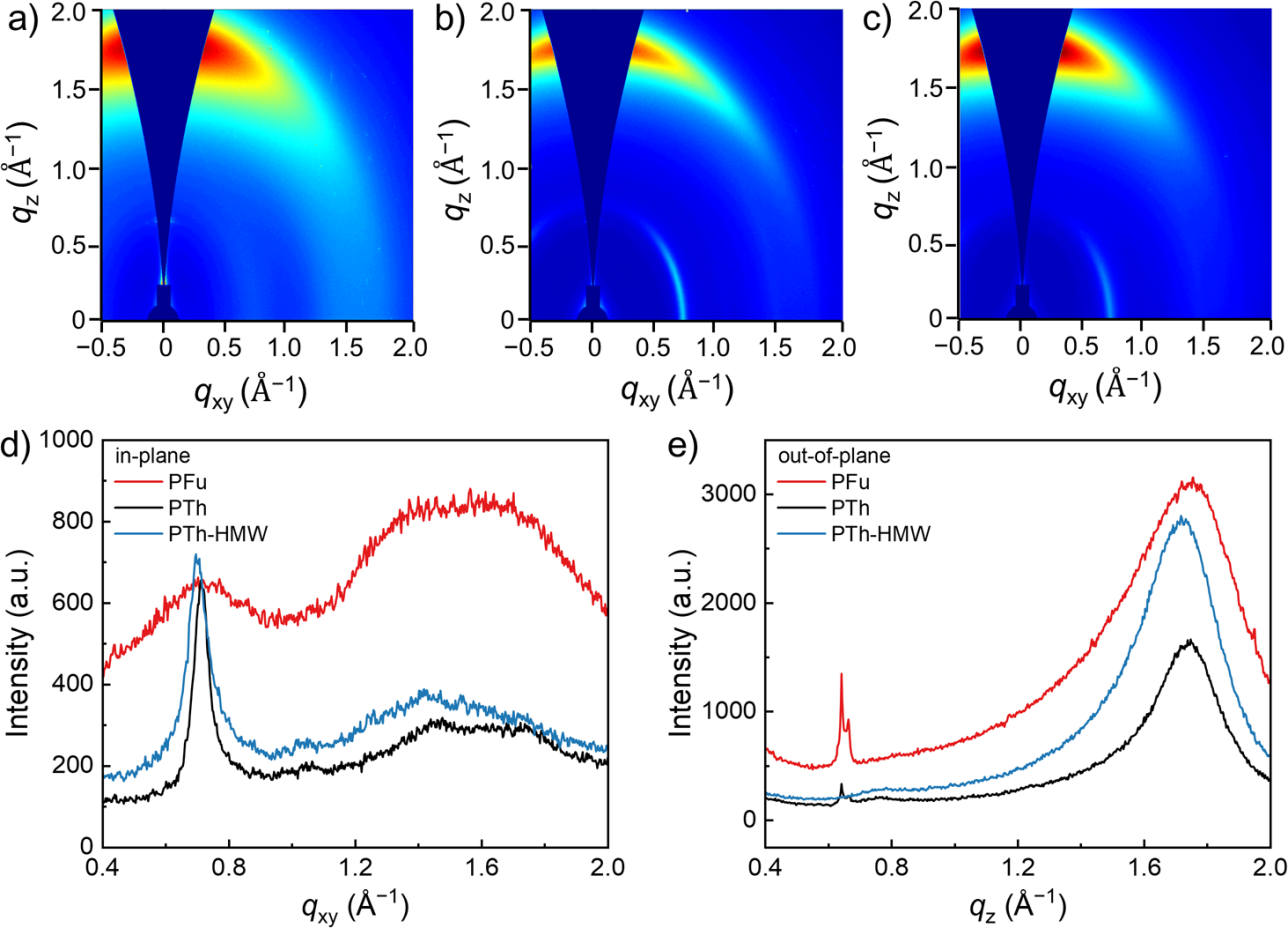
1D and 2D GIWAXS patterns of thin films of (a) PFu, (b) PTh, and (c) PTh-HMW. (d) In-plane and (e) out-of-plane 1D GIWAXS line cuts of the three samples.

In contrast, PFu displayed a much weaker degree of ordering, seen by a weaker and broader (100) lamellar stacking peak, a shorter coherence length, and larger paracrystalline disorder without preferential orientation (Figure 3a,d,e, Table S2 and Figure S6). The (010) π–π stacking peak of PFu located out-of-plane at *q*_*z*_ = 1.730 Å^–1^ (*d*-spacing = 3.632 Å) showed an increased width, which is in line with the reduced order of this sample. Tentatively, we explain the reduction in order of the PFu film by its stronger kinked backbone that results from the different geometries of furan. At the same time, the π–π stacking distance of PFu is smaller than that for PTh, which arises from the likely more coplanar backbone enabled by the smaller dihedral angles of Fu-Ph motifs (Figure 3e).^22,23^ For a more detailed peak analysis, see Figure S6. The sharp peak in the patterns of PFu and also of PTh at *q*_*z*_ = 0.67 Å^–1^ is assigned to an artifact of unknown origin. The PTh-HMW film exhibited a similar GIWAXS pattern with similar order and orientation compared to that of PTh. From the microstructural analysis, we concluded that the heteroatom has a major effect on ordering, with PTh furnishing face-on-oriented films with much better order compared to PFu. The increased chain length of PTh-HMW did not cause major changes to this situation.

Additionally, the thin-film surface was examined by using atomic force microscopy (AFM). Both PTh and PFu exhibited similar surface texture and comparable surface roughness, with root-mean-square (RMS) values of 1.98 and 2.27 nm, respectively (Figure S7). These results indicate that the comonomer unit had a negligible impact on the surface roughness. In contrast, molecular weight has a significant effect. The high-molecular-weight variant, PTh-HMW, displayed a much smoother surface morphology with an RMS roughness of just 0.72 nm.

We next evaluated the electrical characteristics of PFu and PTh in OECTs. The devices were fabricated as described in the Supporting Information, and their performance is summarized in Table 2. Both polymers exhibited typical n-type accumulation-mode transfer (Figure 4a) and output (Figure 4c,d) characteristics. However, the maximum drain current (*I*_D_) (cf. Figure 4a) and the geometry-normalized transconductance (*g*_m,norm_) (Figure 4b) of PTh were 38.59 μA and 33.19 S cm^–1^, respectively, which is approximately 1 order of magnitude higher than those of PFu (3.48 μA and 3.15 S cm^–1^). The difference in performance can be associated with the inferior ordering of films of PFu (cf. Figure 3d). From the transfer curves, we calculated the product of charge-carrier mobility and volumetric capacitance (μC*) to be 116.16 F cm^–1^ V^–1^ s^–1^ for PTh and 10.66 F cm^–1^ V^–1^ s^–1^ for PFu (Figure S8). Notably, the *g*_m__,__norm_ and μC* values of PTh are comparable to those of the previously reported polymer P­(PyV)-H, which contains the bispyridinium phenylene structure,^19^ and they rank among the highest reported values for n-type OECT materials (Figure 4f and Table S1).

**Table 2:** Summarized Results of the OECT Characterization

polymer	*I*_D,max_ /μA	*g*_m,norm_ /S cm^–1^	τ_ON_ /ms	τ_OFF_ /ms	μC*/F cm^–1^ V^–1^ s^–1^	C* /F cm^–3^	μ /cm^2^ V^–1^ s^–1^
PFu	3.48	3.15	0.36	0.30	10.66^a^ 8.74^b^	47	0.23
PTh	38.59	33.19	0.31	0.30	116.16^a^ 108.12^b^	396	0.29
PTh-HMW	72.80	65.87	0.25	0.20	225.71^a^ 213.15^b^	387	0.58

a Extracted from the slope of the *I*_D_^0.5^ vs. *V*_G_ linear fit in Figure S8.

b Extracted from the slope of the *g*_m_ vs. *WdL* ^–1^ (*V*_G_–*V*_th_) linear fit in Figure S10.

**Figure 4:**
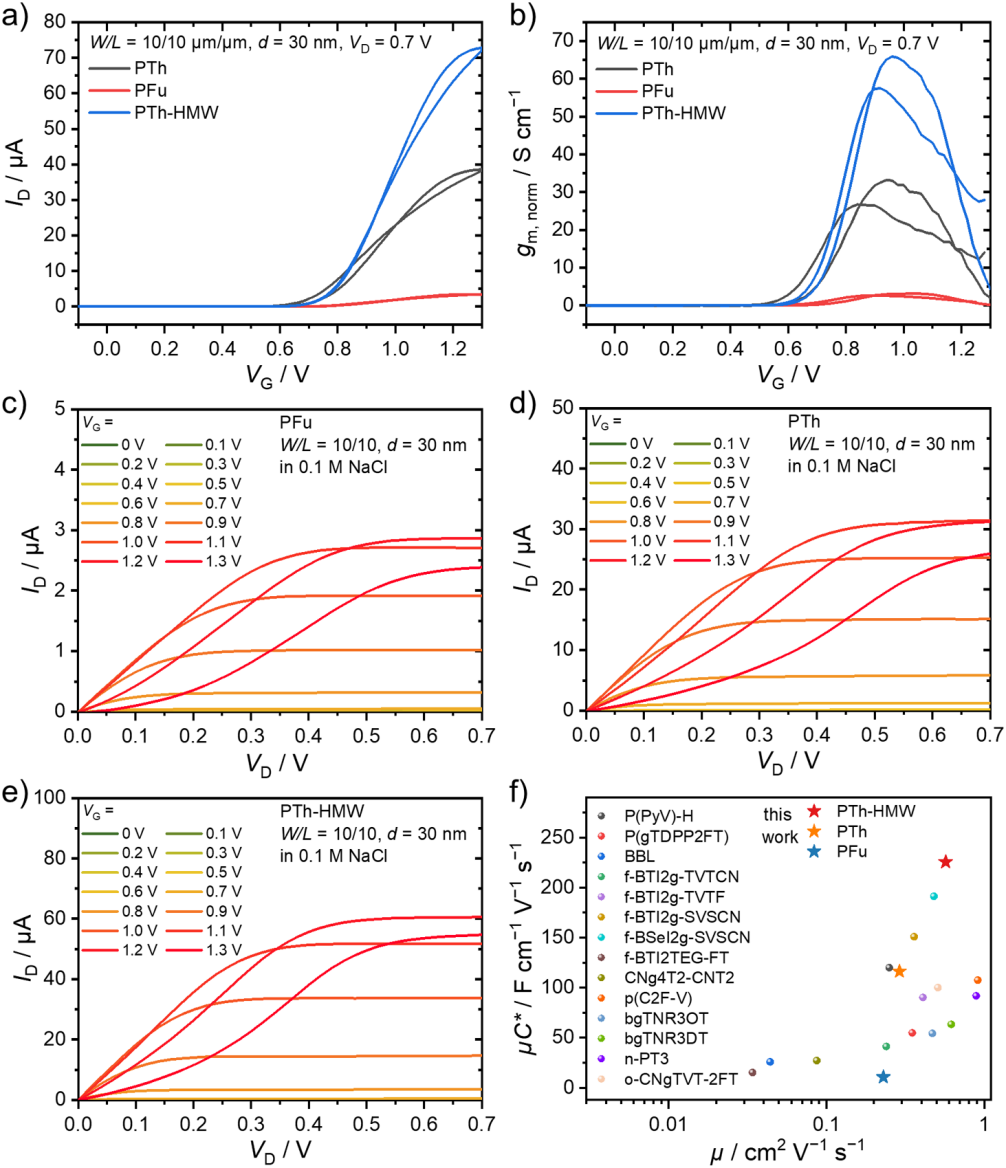
(a) Transfer characteristics and (b) geometry-normalized transconductance of the OECTs. (c–e) Output characteristics of PFu-, PTh-, and PTh-HMW-based OECTs, respectively. All OECTs had the same channel geometry (*W* = 10 μm, *L* = 10 μm, and *d* = 30 nm). (f) Graphical comparison of material performance parameters (μC* vs. μ) with literature data.

To further investigate their electrochemical properties, electrochemical impedance spectroscopy (EIS) was used to determine the volumetric capacitance C* values of both polymers. The results showed C* values of 396 ± 17 F cm^–3^ for PTh and 47 ± 4 F cm^–3^ for PFu (Figure S9), indicating that PTh has superior ion storage and transport capabilities. Using the μC* and C* values, we calculated the electron mobilities μ of PTh and PFu as 0.29 cm^2^ V^–1^ s^–1^ and 0.23 cm^2^ V^–1^ s^–1^, respectively. These values are comparable to state-of-the-art n-type OECT materials (cf. Table S1). Previously, a varied donor strength of the comonomer has been reported to alter ion capacity.^28,29^ However, we also note that the higher water solubility of PFu may contribute to its lower C* as a result of material loss during electrochemical operation, as observed in the spectro-electrochemistry experiments (cf. Figure S5). This would likely lead to an underestimation of the C* and an overestimation of the mobility.

Additionally, both materials exhibited a fast and transient response. PTh-based OECTs demonstrated τ_ON_ = 0.31 ms and τ_OFF_ = 0.30 ms, while PFu-based OECTs showed τ_ON_ = 0.36 ms and τ_OFF_ = 0.30 ms (cf. Figure S8). Given the crucial role of molecular weight in optimizing OECT performance,^17^ we also evaluated the performance of PTh-HMW. As shown in Figure 4b,e,f PTh-HMW exhibited nearly twice the performance of PTh, with an excellent *g*_m,norm_ of 65.87 S cm^–1^ and a record-high μC* of 225.71 F cm^–1^ V^–1^ s^–1^. The improvement in performance is primarily attributed to a significant enhancement in μ of 0.58 cm^2^ V^–1^ s^–1^, while the C* remained largely unchanged. These results represent the best figure of merit for n-type accumulation-mode OECTs (cf. Table 2). Figure 4f compares and highlights the μC* vs. μ performance of the polymers tested herein in comparison to other n-type materials, demonstrating the superior performance of PTh-HMW. The trends in the values of μC* of the three polymers were further confirmed upon variation of the channel geometry, as shown in Figure S10. Finally, OECT device stability was evaluated. While devices made from pristine PTh showed a somewhat better stability (Figure S11), electrostatic cross-linking by immersing the polymer film into a1,3-benzenedisulfonate solution improved the stability of PTh-HMW compared with the other polymers and also the pristine devices (Figure S12). More robust approaches, such as incorporating multinetwork hydrogels^30^ or using acrylate-based cross-linkers,^31^ are potent strategies toward further improvement of long-term operational stability.

## 4 Conclusion

In summary, we have synthesized the n-type copolymer PFu and compared its property profile and OECT performance with its thiophene analogue PTh. The major differences between PFu and PTh were a more tedious synthetic preparation and significantly increased water solubility for the former. Furthermore, in thin films, PFu was mostly amorphous, while PTh showed greater ordering and preferential face-on orientation. The OECT performance of PTh was approximately 1 order of magnitude higher than that of PFu, as indicated by μC* values of 116.16 and 10.66 F cm^–1^ V^–1^ s^–1^, respectively. We also prepared PTh with a higher molecular weight, PTh-HMW, and investigated the impact of chain length on the OECT performance. PTh-HMW demonstrated nearly twice the performance of PTh, achieving an outstanding μC* value of 225.71 F cm^–1^ V^–1^ s^–1^ and a high μ of 0.58 cm^2^ V^–1^ s^–1^, underscoring the critical role of chain length for enhancing device performance. These values represent the highest ever reported for n-type accumulation-mode OECT materials, highlighting their potential for high-performance bioelectronic applications, neuromorphic computing, and next-generation transistor technologies.

## Supplementary Material



## Notes

Detailed information about materials and syntheses; protocols of monomer synthesis, polymerization, and quaternization, including characterization data; overview of the literature; SEC curves; UV–vis spectra; additional ^1^H, ^13^C­{^1^H}, and ^29^Si­{^1^H} NMR spectra (Figures S13–S19); solubility data; spectro-electrochemistry data; GIWAXS peak analysis; AFM images; OECT transfer curves, transient response, and stability; and EIS data (PDF)
