# Supplementary material for: Molar Mass Improves the Performance of n‑Type Organic Electrochemical Transistors
Source: Chem Mater. 2025 Jul 7;37(14):5226–33. doi: 10.1021/acs.chemmater.5c00949 (PMC12288006; doi:10.1021/acs.chemmater.5c00949)
Supplement: Supplementary file 1 [file cm5c00949_si_001.pdf]

Supporting Information for

Molar Mass Improves the Performance of n-Type Organic Electrochemical Transistors

Dominik Stegerer[1,‡], *Tiefeng Liu*[2,3,‡], *Miao Xiong*[2], *Han-Yan Wu*[2], *Min Gyu Kang*[4], *Han Young Woo*[4], *Simone Fabiano*[2,3]*, *Michael Sommer*[1,5]*

[1] Institut für Chemie, Technische Universität Chemnitz, 09111 Chemnitz, Germany.

[2] Laboratory of Organic Electronics, Department of Science and Technology, Linköping University, SE-601 74 Norrköping, Sweden.

[3] Wallenberg Initiative Materials Science for Sustainability, Department of Science and Technology, Linköping University, SE-601 74 Norrköping, Sweden.

[4] Department of Chemistry, College of Science, Korea University, 136-713 Seoul, Republic of Korea.

[5] Center for Materials, Architectures and Integration of Nanomembranes (MAIN), Technische Universität Chemnitz, 09126 Chemnitz, Germany.

[‡] These authors contributed equally.

* Email:

michael.sommer@chemie.tu-chemnitz.de

simone.fabiano@liu.se

Table of Content

Materials, Instruments and Methods. 3

Syntheses 6

Additional Figures and Tables 18

Additional NMR Spectra 29

References 32

Materials, Instruments and Methods.

Materials. All chemicals and materials were purchased from commercial suppliers and used without further purification unless otherwise stated. Dimethoxyethane (DME), petroleum ether (PE) 30/75, and ethyl acetate (EtOAc) were distilled under reduced pressure. Acetonitrile (MeCN), $CHCl_3$:MeCN mixture (1:1 *v*/*v*), *N*,*N*-dimethylformamide (DMF), and 1,4-dioxane were dried over molecular sieves. THF was dried in a circulating distillation apparatus over sodium under argon atmosphere until added benzophenone indicator turned blue-violet and then stored over molecular sieves. 2,5-Bis(trimethylstannyl)thiophene was recrystallized from diethyl ether. 1,4-Dibromo-2,5-bis(bromomethyl)benzene (1),[1] 1,4-dibromo-2,5-divinylbenzene (2),[2] 1,4-dibromo-2,5-bis(2-hydroxyethyl)benzene (3),[3] 1,4-dibromo-2,5-bis(2-(*tert*-butyldimethylsilyloxy)ethyl)benzene (4),[3] 1,4-bis(2-(*tert*-butyldimethylsilyloxy)ethyl)-2,5-bis(4,4,5,5-tetramethyl-1,3,2-dioxaborolan-2-yl)benzene (5),[3,4] 1,4-bis(5-bromopyrid-2-yl)-2,5-bis(2-(*tert*-butyldimethylsilyloxy)ethyl)benzene (PyPhPy),[3] and PymPhPym[3] were synthesised according to modified literature procedures. The synthesis of 2,5-bis(trimethylstannyl)furan is published elsewhere.[5]

Nuclear magnetic resonance (NMR) spectroscopy. NMR spectra were recorded on a Bruker Avance NEO 600 spectrometer (^{1}H: 600.13 MHz, ^{13}C: 150.90 MHz, ^{29}Si: 119.23 MHz). The ^{1}H and ^{13}C NMR spectra were referenced to the residual solvent peak ($CDCl_3$: $\delta(^1H)$ = 7.26 ppm, $\delta(^{13}C)$ = 77.16 ppm; acetone-d_6: $\delta(^1H)$ = 2.05 ppm; DMSO-d_6: $\delta(^1H)$ = 2.50 ppm, $\delta(^{13}C)$ = 39.52 ppm; D_2O: $\delta(^1H)$ = 4.79 ppm, δ(^{13}C, MeOH was used as internal reference here) = 49.50 ppm). The ^{29}Si NMR spectra were referenced on external TMS ($\delta(^{29}Si)$ = 0 ppm). Peak assignments are supported by HSQC, HMBC, and COSY experiments for small molecules; and by COSY experiments for polymers.

Size exclusion chromatography (SEC). SEC of the non-quaternised polymers was measured at 40 °C on a Shimadzu system comprising a 5 µm precolumn and three 5 µm SDV columns with pore sizes ranging from 10^3 to 10^6 Å (Polymer Standards), connected in series with a RID-20A RI detector and a SPD-20AV UV-vis detector (Shimadzu) using relative calibration with polystyrene standards. THF was used as eluent with a flow rate of 1 mL/min.

Polymer processing. Generally, quaternised polymers were dissolved at RT in 2,2,2-trifluoroethanol (TFE), and solutions were stirred overnight. Elevated temperatures were used for other solvents (e.g. D_2O) when necessary.

Cyclic voltammetry (CV). CV measurements of polymer films were performed with a scan rate of 50 mV·s^{-1} at RT under argon atmosphere using a PalmSens4 potentiostat, a Ag/Ag$^+$ pseudo-reference electrode, ITO-coated float glass (from Präzisions Glas & Optik GmbH (pgo), sheet resistance ≤7 Ω·square^{-1}) as working electrode, and a platinum wire as counter electrode. The supportive electrolyte was NBu_4PF_6 (0.1 M) in dry acetonitrile. Ferrocene was used for calibration, and a value of −4.80 eV for the half-wave potential of the Fc/Fc$^+$ couple was used for determining the LUMO energy levels. The error of the redox potentials determined from the CV curves via the onset method is estimated as ±0.04 V, while the error of the LUMO energy levels is estimated to be ±0.10 eV.[6] Thin films were prepared by spin coating (1000 rpm, 60 s) polymer solutions (5 g/L in TFE, 4 µL) onto the ITO electrodes.

UV-vis absorption spectroscopy. The absorption spectra of solutions were measured on an Agilent Cary 60 UV-vis spectrophotometer at 25 °C in a 10 mm cuvette. The absorption spectra of thin films were recorded on a Flame-S UV-vis spectrometer from Ocean Optics at 25 °C using OceanView 1.5.2 software. Thin films were prepared by spin coating (1000 rpm, 60 s) polymer solutions (5 g/L in TFE, 10 µL) onto quartz glass substrates.

Spectro-electrochemistry. Measurements were carried out at RT under argon in a homemade spectro-electrochemistry cell consisting of a 10 mm quartz glass cuvette and a PTFE cap for fixing the electrodes. The electrochemistry setup consisted of a PalmSens4 potentiostat, an aqueous Ag/AgCl reference electrode, an ITO-coated float glass (from Präzisions Glas & Optik GmbH (pgo), sheet resistance ≤7 Ω·sq^{-1}) as working electrode, and a platinum wire as counter electrode. The supportive electrolyte was NaCl (0.1 M) in water. Polymer thin films were prepared by spin coating (1000 rpm, 60 s) the polymer solutions (2 g/L in TFE, 10 µL) onto the ITO electrode. UV−vis spectra were recorded with a Cary 60 UV−vis spectrophotometer (Agilent Technologies) after the application of fixed potentials for 100 s each. Corresponding CV measurements were performed with a scan rate of 50 mV·s^{-1}.

OECT fabrication and characterisation. OECTs were fabricated following a previously reported procedure.[7] In detail, microscope glass slides were thoroughly cleaned via successive sonication in soap water, deionized water, acetone and isopropanol, followed by N_2 drying. Source and drain electrodes were thermally evaporated with 5 nm Cr and 50 nm Au and patterned with photolithography and wet etching. A first layer of parylene C (1 µm) was coated along with a drop of 3-(trimethoxysilyl)propyl methacrylate, to serve as an insulating layer

between the metal electrode and the electrolyte. Next, a 2% Micro-90 surfactant solution was spin-coated, followed by deposition of a second sacrificial parylene C (1 μm). To protect parylene C layers during a subsequent plasma reactive ion etching step, a thick positive photoresist (5 μm, AZ 10XT 520CP) was spin-coated on top of the parylene C. A second photolithographic patterning step followed by an application of AZ developer was performed to define the contact pads and the OECT channels. A plasma reactive ion etching step (150 W, O_2 = 500 sccm, CF_4 = 1000 sccm, 380 s) was then applied to remove the organic layers including photoresist and parylene C, exposing the contact pad and OECT channel areas while keeping other parts of the device covered with two layers of parylene C. The channel between source and drain was patterned to be W/L = 10 μm/10 μm. The OECT devices were spin-coated with PFu, PTh, and PTh-HMW solutions (5 mg ml^{-1} in TFE) at 1000 rpm to form polymer films of approximately 30 nm. The thickness was measured using a stylus profilometer (Dektak XT, Bruker). The sacrificial parylene C was then peeled off, removing the polymer film on top of it and leaving the polymer in the patterned area corresponding to the OECT channel and the electrode pads. For all OECTs, aqueous 0.1 M NaCl solution was used as the electrolyte, and an Ag/AgCl pellet (purchased from VWR) served as the gate electrode. The devices were characterized using a Keithley 4200A-SCS instrument with a 4225-PMU Ultra Fast I-V Module and 4225-RPM Remote Amplifier/Switch Modules. Electrostatic crosslinking was performed by immersing films in a solution of 1,3-benzenedisulfonate in dimethyl sulfoxide (5 mg/mL) for 5 minutes, followed by rinsing with water.

Electrochemical impedance spectroscopy (EIS). EIS was performed on a BioLogic SP-200 potentiostat. Aqueous 0.1 M NaCl solution was used as the electrolyte, a platinum wire was used as the counter electrode (CE), a Ag/AgCl electrode was used as the reference electrode (RE), and patterned polymer thin films with a volume of 0.77×10^{-7} to 7.5×10^{-7} cm^3 on Cr/Au electrodes on glass substrates served as the working electrode (WE). The WE was set to −0.7 V for volumetric capacitance measurements, and spectra were recorded in the frequency range of 1 Hz–100 kHz. The capacitance was extracted by fitting to the modified Randles circuit model: $R_s + R_p$/CPE, where R_s is the active electrolyte resistance, CPE is a constant phase element and R_p is the charge transfer resistance.

Grazing-incidence wide-angle X-ray scattering (GIWAXS). The samples were spin-coated on silicon wafers in a glovebox for characterisation. Measurements were performed at the 9A U-SAXS beamline of the Pohang Accelerator Laboratory (PAL), Republic of Korea, with a

beam energy of 11.07 keV and incident angles of 0.12°. The paracrystallinity disorder (g) was estimated using the equation $g^2 = \Delta q/2\pi q_o$, where q_o is the peak position and Δq is the full width at half maximum of the peak. The coherence length (L_c) was calculated from $L_c = 2\pi K/\Delta q$, where K is the coefficient typical take as ~ 0.9.[8]

Atomic force microscopy (AFM). The materials were spin-coated on silicon wafers for morphology characterisation using a Bruker Dimension Icon XR. The AFM images were acquired in tapping mode with a silicon nitride cantilever having a spring constant of 40 N m^{-1}.

Syntheses

Synthetic remarks. The procedure[2] for the Wittig olefination of 1 was found to be reproducible, and slightly higher yields were obtained (Scheme S1). The final recrystallization step was omitted due to the high purity of 2 after the silica plug. Water-free reaction conditions were found to be crucial to achieve a quantitative yield of the silylation reaction. For this purpose, dialcohol 3 was ground under vacuum with a magnetic stirring bar, and a solution of imidazole in DMF was dried over molecular sieves. Full conversion of the reaction was achieved after the reaction mixture was shortly heated to 80 °C until solid particles got molten or dissolved. The yield of the borylation of 4 was significantly increased by changing the solvent from DMSO to 1,4-dioxane. The final recrystallization was again omitted in this step due to the high purity of 5 after column chromatography. To the Suzuki reaction of 5 with 2,5-dibromopyridine, a purification step by column chromatography was added, and the product PyPhPy was recrystallized from DME two to three times until impurities were no longer visible. PyPhPy was obtained in higher yield compared to published reports.[3,9–11]

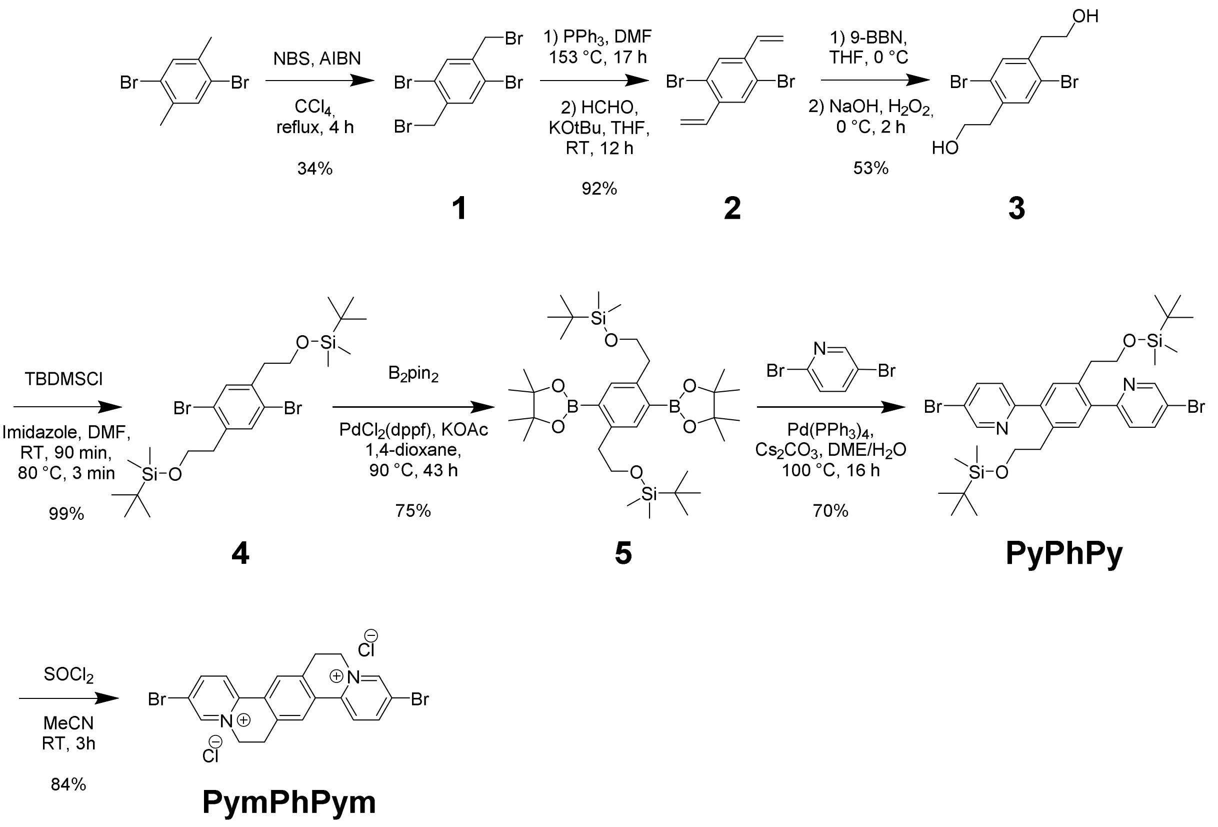

Scheme S1. Synthetic route to the monomer PyPhPy and the model compound PymPhPym.

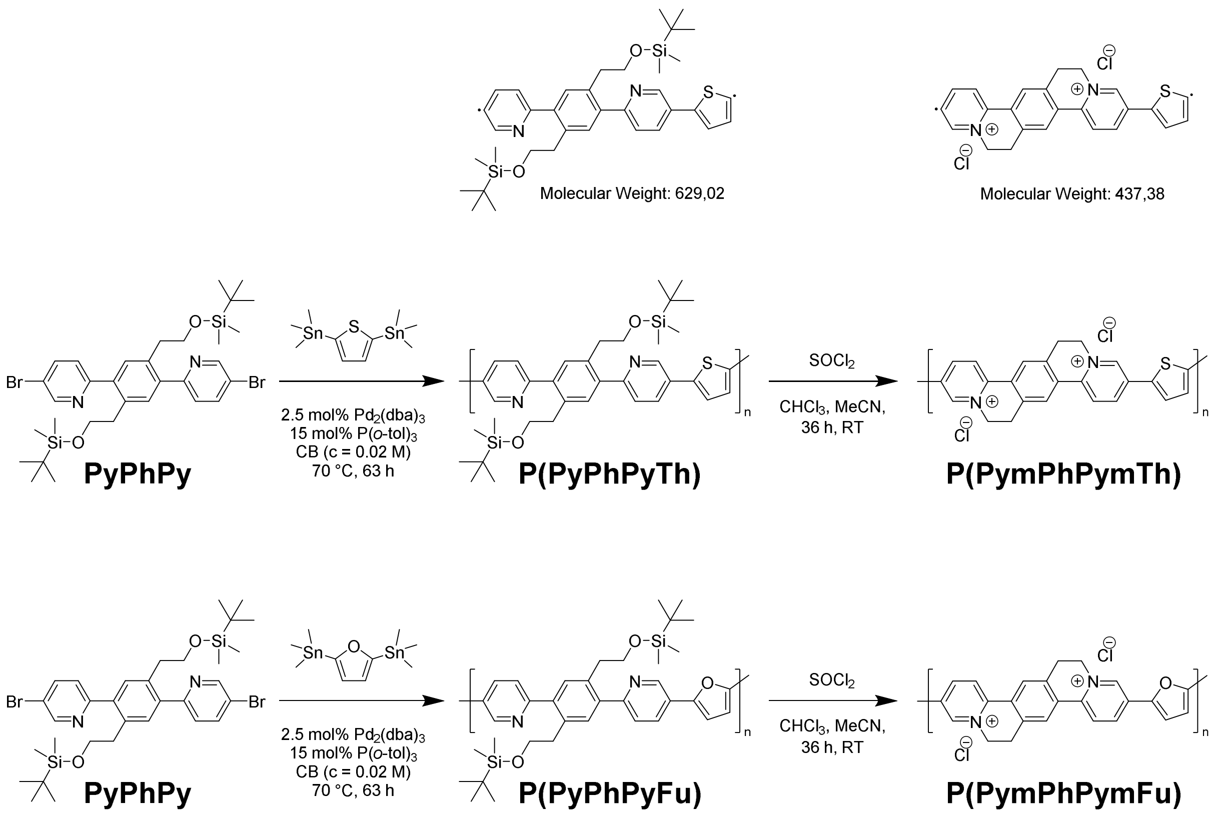

Scheme S2. Polymer synthesis and subsequent quaternisation.

Synthesis of 1,4-dibromo-2,5-bis(bromomethyl)benzene *(1)*.

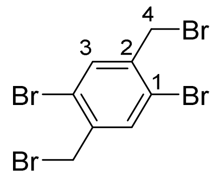

A 1 L two-neck round bottom flask was charged with 1,4-dibromo-2,5-dimethylbenzene (21.12 g, 80.01 mmol), *N*-bromosuccinimide (NBS) (28.49 g, 160.1 mmol, 2.0 eq), and 2,2′-azobis(isobutyronitrile) (AIBN) (250 mg, 1.52 mmol, 0.02 eq) under argon atmosphere. CCl_4 (320 mL) was added. The white suspension was additionally degassed with argon for 1 min and then heated to reflux for 4 h. The warm mixture was filtered, the residue was washed with PE 30/75, and the solvent was evaporated under reduced pressure from the filtrate. The crude mixture was recrystallized once from methanol/chloroform (7:16 *v*/*v*) yielding 1 as slightly yellowish crystals (11.74 g, 27.84 mmol, 34%). T_m = 158–160 °C.

^{1}H NMR ($CDCl_3$, 600 MHz, 25 °C): δ = 7.66 (s, 2H, H_3), 4.51 (s, 4H, H_4) ppm. ^{13}C{^{1}H} NMR ($CDCl_3$, 151 MHz, 25 °C): δ = 139.13 (C_2), 135.50 (C_3), 123.43 (C_1), 31.63 (C_4) ppm.

Synthesis of 1,4-dibromo-2,5-divinylbenzene *(2)*.

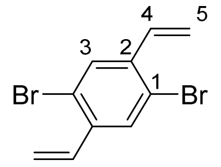

In a 250 mL two-neck round bottom flask, a solution of 1,4-dibromo-2,5-bis(bromomethyl)-benzene (5.853 g, 13.88 mmol) and triphenylphosphine (9.105 g, 34.71 mmol, 2.5 eq) in DMF (70 mL) was heated to reflux for 17 h, and a white precipitate was formed. The solvent was evaporated under reduced pressure, the atmosphere was changed to argon, and paraformaldehyde (8.970 g, 298.7 mmol, 22 eq) and dry THF (110 mL) were added. A suspension of potassium *tert*-butoxide (4.704 g, 41.92 mmol, 3.0 eq) in dry THF (20 mL) was transferred into the reaction mixture, and the colour turned quickly into orange, then to yellowish white. The suspension was stirred at RT overnight, then, the solvent was evaporated under reduced pressure. The residue was extracted 4 x with PE 30/75, and the extracts were filtered through a silica plug yielding 2, after evaporation of the solvent under reduced pressure, as a yellowish white solid (3.69 g, 12.8 mmol, 92%). T_m = 79–82 °C.

^{1}H NMR ($CDCl_3$, 600 MHz, 25 °C): δ = 7.72 (s, 2H, H_3), 6.95 (dd, $^3J_{trans}$ = 17.4 Hz, $^3J_{cis}$ = 11.0 Hz, 2H, H_4), 5.71 (dd, $^3J_{trans}$ = 17.4 Hz, ^2J = 0.6 Hz, 2H, trans-H_5), 5.40 (dd, $^3J_{cis}$ = 11.0 Hz, ^2J = 0.6 Hz, 2H, cis-H_5) ppm. $^{13}C\{^1H\}$ NMR ($CDCl_3$, 151 MHz, 25 °C): δ = 138.24 (C_2), 134.49 (C_4), 130.67 (C_3), 122.63 (C_1), 117.89 (C_5) ppm.

Synthesis of 1,4-dibromo-2,5-bis(2-hydroxyethyl)benzene *(3)*.

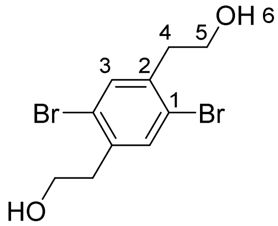

In a 500 mL three-neck round bottom flask, a 9-borabicyclo[3.3.1]nonane (9-BBN) solution (0.5 M in THF, 60 mL, 30 mmol, 2.4 eq) was added dropwise at 0 °C within 30 min to a solution of 1,4-dibromo-2,5-divinylbenzene (3.60 g, 12.5 mmol) in dry and degassed THF (50 mL) under argon atmosphere. The turbid solution was stirred further at RT for 21 h. The white suspension was cooled to 0 °C and quenched by the dropwise addition of MeOH (20 mL). Aqueous NaOH (2 M, 100 mL) and aqueous H_2O_2 (33%, 20 mL) were added carefully, and the mixture was stirred further at RT for 2 h. Saturated aqueous $Na_2S_2O_3$ solution (50 mL) was added, the phases were separated, and the aqueous phase was extracted with EtOAc (4 x 30 mL). The combined organic phases were washed with brine (50 mL) and dried over Na_2SO_4. The solvent was evaporated under reduced pressure, and the resulting oil was purified by column chromatography (SiO_2, PE 30/75:EtOAc, gradient from 3:1 *v*/*v* to pure EtOAc) to yield 3 as a white solid (2.15 g, 6.63 mmol, 53%). T_m = 134–136 °C.

^{1}H NMR (DMSO-d_6, 600 MHz, 25 °C): δ = 7.55 (s, 2H, H_3), 4.74 (t, J = 5.3 Hz, 2H, H_6), 3.59 (td, J = 6.7 Hz, J = 5.4 Hz, 4H, H_5), 2.80 (t, J = 6.8 Hz, 4H, H_4) ppm. $^{13}C\{^1H\}$ NMR (DMSO-d_6, 151 MHz, 25 °C): δ = 138.51 (C_2), 134.50 (C_3), 122.80 (C_1), 59.93 (C_5), 38.18 (C_4) ppm.

Synthesis of 1,4-dibromo-2,5-bis(2-(*tert*-butyldimethylsilyloxy)ethyl)benzene *(4)*.

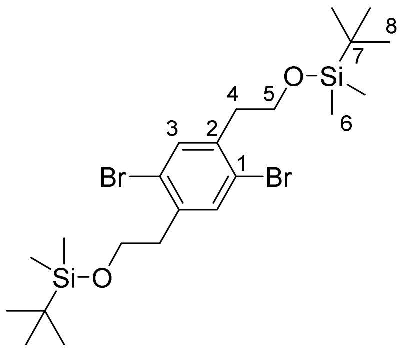

In a 100 mL two-neck round bottom flask, a dry solution of imidazole in DMF (2 M, 22.2 mL, 44.4 mmol, 4 eq) was added to a solution of 3 (3.68 g, 11.4 mmol) in dry DMF (15 mL) under argon atmosphere. The solution was stirred at 0 °C, and *tert*-butyldimethylsilyl chloride (TBDMSCl) (4.0 g, 27 mmol, 2.3 eq) was added. The mixture solidified and then was stirred at RT for 90 min and got molten again. The suspension was heated to 80 °C for 3 min forming an emulsion. The mixture was diluted with DCM (180 mL), washed with H_2O (3 x 60 mL) and brine (60 mL), and dried over Na_2SO_4. The solvent was evaporated under reduced pressure, and the resulting crystals were purified by column chromatography (SiO_2, PE 30/75, then PE 30/75:EtOAc, gradient from 20:1 to 10:1 *v*/*v*) to yield 4 as a colourless crystalline solid (6.23 g, 11.3 mmol, 99%). T_m = 50–51 °C.

^{1}H NMR ($CDCl_3$, 600 MHz, 25 °C): δ = 7.43 (s, 2H, H_3), 3.79 (t, J = 6.7 Hz, 4H, H_5), 2.89 (t, J = 6.7 Hz, 4H, H_4), 0.87 (s, 18H, H_8), −0.02 (s, 12H, H_6) ppm. $^{13}C\{^1H\}$ NMR ($CDCl_3$, 151 MHz, 25 °C): δ = 138.39 (C_2), 135.28 (C_3), 123.11 (C_1), 62.25 (C_5), 38.95 (C_4), 26.04 (C_8), 18.41 (C_7), −5.31 (C_6) ppm. $^{29}Si\{^1H\}$ NMR ($CDCl_3$, 119 MHz, 25 °C): δ = 19.67 ppm.

Synthesis of 1,4-bis(2-(*tert*-butyldimethylsilyloxy)ethyl)-2,5-bis(4,4,5,5-tetramethyl-1,3,2-dioxaborolan-2-yl)benzene *(5)*.

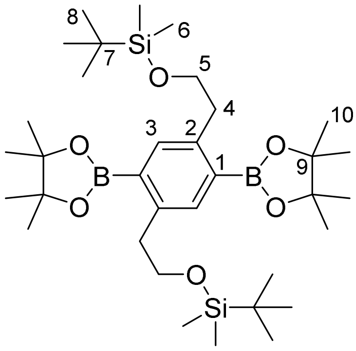

In a 25 mL Schlenk flask, finely pestled potassium acetate (1.173 g, 11.95 mmol, 6.6 eq) was dried under vacuum at 270 °C. After cooling to RT, compound 4 (1.002 g, 1.813 mmol), [1,1′-bis(diphenylphosphino)ferrocene-κ^2*P*,*P*′]dichloropalladium(II) ($PdCl_2$(dppf)) (80.2 mg, 110 µmol, 6 mol%), bis(pinacolato)diboron (B_2Pin_2) (1.102 g, 4.338 mmol, 2.4 eq), and dry and degassed 1,4-dioxane (9 mL) were added under argon atmosphere. The orange-brown suspension was stirred at 90 °C for 43 h. The dark mixture was filtered through kieselguhr (rinsed with EtOAc), and the solvent was evaporated under reduced pressure. The crude product was purified by column chromatography (SiO_2, PE 30/75, then PE 30/75:EtOAc, gradient from 40:1 to 10:1 *v*/*v*) to yield 5 as a white crystalline solid (882 mg, 1.36 mmol, 75%). T_m = 139–140 °C.

^{1}H NMR ($CDCl_3$, 600 MHz, 25 °C): δ = 7.63 (s, 2H, H_3), 3.74 (t, J = 7.4 Hz, 4H, H_5), 3.09 (t, J = 7.4 Hz, 4H, H_4), 1.33 (s, 24H, H_{10}), 0.87 (s, 18H, H_8), −0.02 (s, 12H, H_6) ppm. ^{13}C{^{1}H} NMR ($CDCl_3$, 151 MHz, 25 °C): δ = 142.17 (C_2), 138.10 (C_3), 131.02 (br., C_1), 83.58 (C_9), 65.73 (C_5), 38.91 (C_4), 26.17 (C_8), 24.98 (C_{10}), 18.48 (C_7), −5.11 (C_6) ppm. ^{29}Si{^{1}H} NMR ($CDCl_3$, 119 MHz, 25 °C): δ = 18.24 ppm.

Synthesis of 1,4-bis(5-bromopyrid-2-yl)-2,5-bis(2-(*tert*-butyldimethylsilyloxy)ethyl)benzene *(PyPhPy)*.

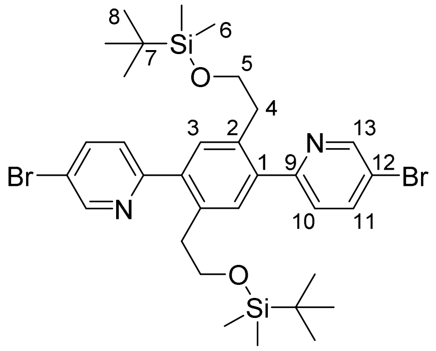

A 55 mL Schlenk tube was charged with 5 (999.9 mg, 1.546 mmol), 2,5-dibromopyridine (1.4640 g, 6.1801 mmol, 4.0 eq), caesium carbonate (2.5205 g, 7.7359 mmol, 5.0 eq), and tetrakis(triphenylphosphine)palladium(0) (177.5 mg, 153.6 µmol, 10 mol%) under argon atmosphere. Degassed DME (16 mL) and degassed H_2O (8 mL) were added, and the suspension was stirred at 100 °C for 16 h forming a two-phase system. After cooling to RT, the mixture was diluted with DCM (40 mL), and the phases were separated. The organic phase was washed with H_2O (2 x 20 mL), the combined aqueous phases were back-extracted with DCM (20 mL), and the combined organic phases were washed with brine (20 mL) and dried over Na_2SO_4. The solvent was evaporated under reduced pressure. The crude product was purified by column chromatography (SiO_2, PE 30/75:EtOAc, gradient from 20:1 to 10:1 *v*/*v*) using the dry-load method on silica (5 g) and afterwards recrystallized from DME to yield PyPhPy as colourless crystals (772.8 mg, 1.094 mmol, 70%). T_m = 154–155 °C.

^{1}H NMR ($CDCl_3$, 600 MHz, 25 °C): δ = 8.75 (d, ^4J = 2.2 Hz, 2H, H_{13}), 7.88 (dd, ^3J = 8.3 Hz, ^4J = 2.4 Hz, 2H, H_{11}), 7.37 (d, ^3J = 8.3 Hz, 2H, H_{10}), 7.32 (s, 2H, H_3), 3.71 (t, J = 7.4 Hz, 4H, H_5), 2.94 (t, J = 7.4 Hz, 4H, H_4), 0.80 (s, 18H, H_8), −0.08 (s, 12H, H_6) ppm. ^{13}C{^{1}H} NMR ($CDCl_3$, 151 MHz, 25 °C): δ = 158.18 (C_9), 150.41 (C_{13}), 139.80 (C_1), 139.03 (C_{11}), 135.10 (C_2), 132.48 (C_3), 125.57 (C_{10}), 119.43 (C_{12}), 64.38 (C_5), 36.26 (C_4), 26.04 (C_8), 18.45 (C_7), −5.26 (C_6) ppm. ^{29}Si{^{1}H} NMR ($CDCl_3$, 119 MHz, 25 °C): δ = 19.25 ppm.

Synthesis of PymPhPym.

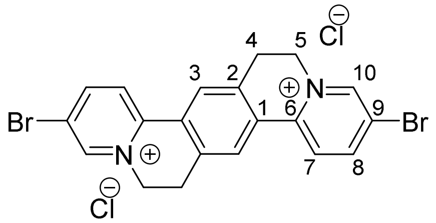

In a 30 mL Schlenk tube, PyPhPy (49 mg, 69 µmol) was suspended in dry MeCN (7.5 mL) under argon atmosphere. An excess of $SOCl_2$ (1.5 mL, 2.4 g, 21 mmol, 300 eq) was added, and the colourless suspension was stirred at RT for 3 h becoming a pale yellow suspension. The volatiles were removed via sublimation at low temperature and pressure, and the collected residue was washed with a tiny amount of H_2O yielding PymPhPym as a yellow-beige solid (30 mg, 58 µmol, 84%). T_m > 300 °C.

^{1}H NMR (D_2O, 600 MHz, 25 °C): δ = 9.18 (d, ^4J = 1.4 Hz, 2H, H_{10}), 8.80 (dd, ^3J = 8.8 Hz, ^4J = 1.8 Hz, 2H, H_8), 8.51 (d, ^3J = 8.8 Hz, 2H, H_7), 8.23 (s, 2H, H_3), 4.88 (t, J = 6.7 Hz, 4H, H_5), 3.46 (t, J = 6.7 Hz, 4H, H_4) ppm. ^{13}C{^{1}H} NMR (D_2O, 151 MHz, 25 °C): δ = 149.28 (C_8), 147.42 (C_6), 147.10 (C_{10}), 136.25 (C_2), 130.24 (C_1), 127.55 (C_3), 126.37 (C_7), 121.60 (C_9), 55.98 (C_5), 26.37 (C_4) ppm.

General polymerisation procedure.

All polymerisations were identical except for monomer stoichiometry as specified below. A 15 mL screw cap vial was charged with the stannylated comonomer, PyPhPy (1.00 eq), $Pd_2(dba)_3 \cdot CHCl_3$ (2.5 mol%), and tri(*o*-tolyl)phosphine (15 mol%) under argon atmosphere. Dry and degassed chlorobenzene (c = 0.02 M, calc. on PyPhPy) was added, the vial was sealed with PTFE tape and covered with aluminium foil to exclude light, and the red mixture was stirred at 70 °C for 63 h. The yellow mixture was concentrated until gelation occurred, then diluted with $CHCl_3$ (~1 mL), and precipitated in MeOH. The precipitate was collected by filtration, washed with MeOH, and dried at RT under vacuum yielding the desired polymer.

Synthesis of P(PyPhPyFu).

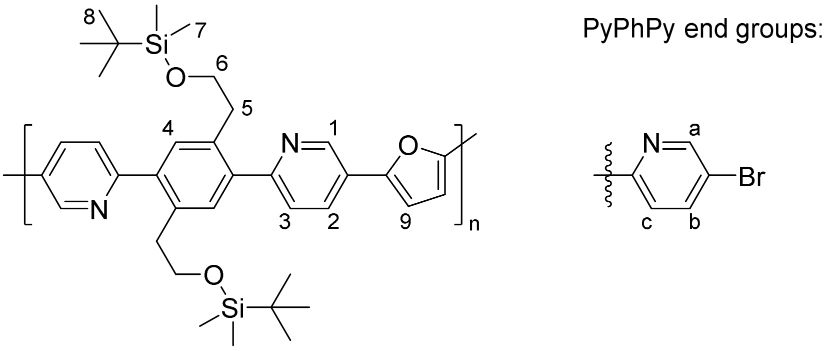

PyPhPy (116.71 mg, 165.03 µmol), 2,5-bis(trimethylstannyl)furan (61.70 mg, 156.7 µmol, 0.95 eq), $Pd_2(dba)_3 \cdot CHCl_3$ (4.32 mg, 4.17 µmol, 2.5 mol%), tri(*o*-tolyl)phosphine (7.54 mg, 24.8 µmol, 15 mol%), chlorobenzene (8.2 mL).

P(PyPhPyFu) was obtained as a greenish yellow solid (96.1 mg, 95%). ^1H NMR ($CDCl_3$, 600 MHz, 25 °C): δ = 9.13 (s, 2H, H_1), 8.77 (br. d, 1H, H_a), 8.13 (br. d, 2H, H_2), 7.90 (br. dd, 1H, H_b), 7.57 (br. d, 2H, H_3), 7.44 (s, 2H, H_4), 7.40 (d, 1H, H_c), 6.96 (br, 2H, H_9), 3.80 (br. t, 4H, H_6), 3.07 (br, 4H, H_5), 0.83 (s, 18H, H_8), −0.04 (s, 12H, H_7) ppm. The number of protons are with respect to the repeat unit, except for the end-group signals. Molecular weight from NMR end-group analysis: DP_n = 19, M_n = 12 kg/mol. Molecular weight from SEC in THF: M_n = 12.4 kg/mol, M_w = 25.1 kg/mol, $Đ$ = 2.0.

Synthesis of P(PyPhPyTh).

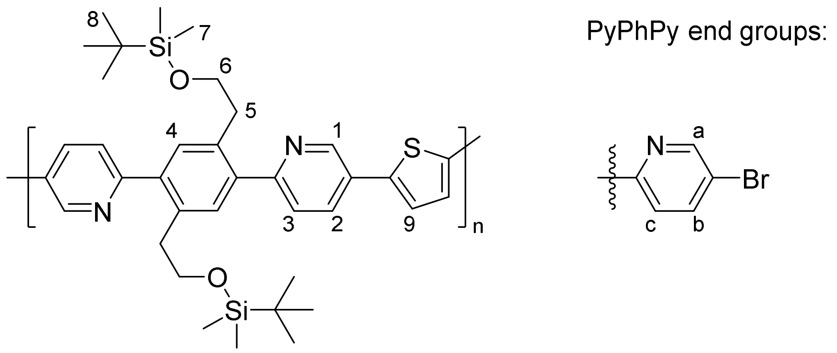

PyPhPy (199.92 mg, 282.69 µmol), 2,5-bis(trimethylstannyl)thiophene (106.62 mg, 260.03 µmol, 0.92 eq), $Pd_2(dba)_3 \cdot CHCl_3$ (7.32 mg, 7.07 µmol, 2.5 mol%), tri(*o*-tolyl)phosphine (13.02 mg, 42.78 µmol, 15 mol%), chlorobenzene (14.0 mL).

P(PyPhPyTh) was obtained as a light yellow solid (181.0 mg, 96%). ^{1}H NMR ($CDCl_3$, 600 MHz, 25 °C): δ = 9.04 (s, 2H, H_1), 8.77 (d, 1H, H_a), 8.01 (br, 2H, H_2), 7.90 (dd, 1H, H_b), 7.56 (br, 2H, H_3), 7.49 (br, 2H, H_9), 7.43 (s, 2H, H_4), 7.40 (d, 1H, H_c), 3.80 (br, 4H, H_6), 3.07 (br, 4H, H_5), 0.83 (s, 18H, H_8), −0.04 (s, 12H, H_7) ppm. The number of protons are with respect to the repeat unit, except for the end-group signals. Molecular weight from NMR end-group analysis: DP_n = 22, M_n = 14 kg/mol. Molecular weight from SEC in THF: M_n = 21.0 kg/mol, M_w = 50.8 kg/mol, $Đ$ = 2.4.

Synthesis of P(PyPhPyTh)-HMW.

PyPhPy (49.97 mg, 70.66 µmol), 2,5-bis(trimethylstannyl)thiophene (28.98 mg, 70.68 µmol, 1.000 eq), $Pd_2(dba)_3 \cdot CHCl_3$ (1.81 mg, 1.75 µmol, 2.5 mol%), tri(*o*-tolyl)phosphine (3.17 mg, 10.4 µmol, 15 mol%), chlorobenzene (3.5 mL). Reaction time was 67 h. The yellow mixture was diluted with $CHCl_3$ (~1 mL) and precipitated in MeOH. The precipitate was collected, washed with MeOH, dried at RT and re-dissolved in $CHCl_3$ (10 mL). The chloroform solution was extracted with aqueous EDTA · 2 Na · 2 H_2O solution (0.2 M, 40 mL, 8 mmol, 3.0 g) overnight at 63 °C. The phases were separated, and the organic phase was washed with H_2O (2 x 15 mL), concentrated, and precipitated again in MeOH. The precipitate was collected by filtration and purified by subsequent Soxhlet extraction with acetone, EtOAc, PE 30/75 and $CHCl_3$. The solvent was evaporated under reduced pressure from the $CHCl_3$ solution containing the polymer, and the residue was dried at 40 °C in a vacuum oven overnight yielding the desired polymer. P(PyPhPyTh)-HMW was obtained as a yellow-orange solid (41.3 mg, 92%). Molecular weight from SEC in THF: M_n = 52.2 kg/mol, M_w = 117 kg/mol, $Đ$ = 2.2.

General quaternisation procedure.

In a screw cap vial, the non-quaternised polymer was dissolved/suspended in a dry mixture of $CHCl_3$ and MeCN (1:1 *v*/*v*) under argon atmosphere. An excess of $SOCl_2$ (300 eq) was added, and the suspension was stirred at RT for 36 h becoming a red and turbid suspension. The volatiles were removed, and the residue was washed with $CHCl_3$ (3 x 5 mL) and dried at RT under vacuum yielding the quaternised polymer.

Synthesis of PFu.

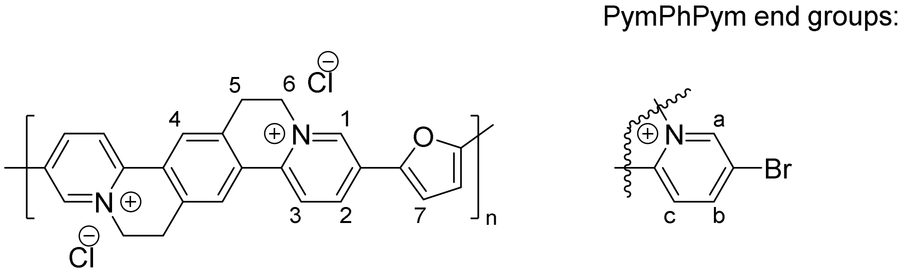

P(PyPhPyFu) (69.8 mg, ~114 µmol), $CHCl_3$:MeCN (1:1 *v*/*v*, 12 mL), $SOCl_2$ (2.5 mL, 4.1 g, 34 mmol, 300 eq).

PFu was obtained as a red solid with a pale greenish shine (58.5 mg). ^1H NMR (D_2O, 600 MHz, 25 °C): δ = 9.30–9.95 (br, 2H, H_1), 9.20 (br. s, 1H, H_a), 8.78–9.15 (br, 2H, H_2), 8.80 (br. d, 1H, H_b), 8.43–8.78 (br, 2H, H_3), 8.52 (br. d, 1H, H_c), 7.90–8.43 (br, 2H, H_4), 7.30–7.90 (br, 2H, H_7), 4.5–5.7 (br, 4H, H_6), 3.0–4.1 (br, 4H, H_5) ppm.

Synthesis of PTh.

PymPhPym end groups:

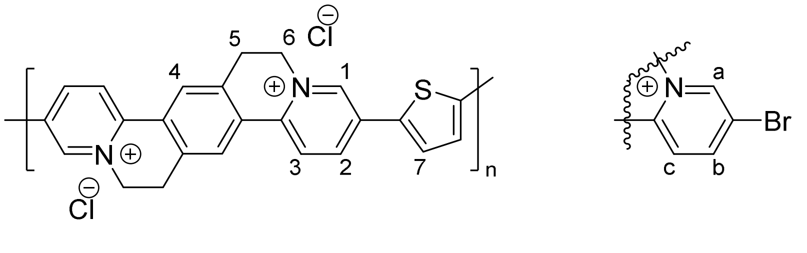

P(PyPhPyTh) (140.0 mg, ~222.6 µmol), $CHCl_3$:MeCN (1:1 *v*/*v*, 24 mL), $SOCl_2$ (5.0 mL, 8.2 g, 69 mmol, 300 eq).

PTh was obtained as a red solid with a pale greenish shine (121.6 mg). ^{1}H NMR (D_2O, 600 MHz, 25 °C): δ = 9.38 (br, 2H, H_1), 9.20 (br, 1H, H_a), ~8.84 (br, 2H, H_2), 8.81 (br, 1H, H_b), ~8.53 (br, 2H, H_3), 8.52 (br, 1H, H_c), 8.24 (br, 2H, H_4), 7.98 (br, 2H, H_7), 4.98 (br, 4H, H_6), 3.54 (br, 4H, H_5) ppm.

Synthesis of PTh-HMW.

P(PyPhPyTh)-HMW (20.0 mg, ~31.8 µmol), $CHCl_3$:MeCN (1:1 *v*/*v*, 3.4 mL), $SOCl_2$ (0.70 mL, 1.1 g, 9.6 mmol, 300 eq). Reaction time was 24 h. The polymer was washed with MeOH (2 mL), H_2O (2 mL), and DMSO (2 mL). PTh-HMW was obtained as a dark red solid with a pale greenish shine (18.2 mg).

Additional Figures and Tables

Table S1. Summary of n-type accumulation-mode OECT performance.

polymer	V_{th} / V	$g_{m, norm}$ / S cm^{-1}	τ_{ON} / ms	τ_{OFF} / ms	I_{ON}/I_{OFF}	μC^* / F cm^{-1} V^{-1} s^{-1}	C^* / F cm^{-3}	μ / cm^2 V^{-1} s^{-1}	REF
PTh-HMW	0.67	65.87	0.25	0.20	4×10^3	225.71[a]<br>213.15[b]	387	0.58	This work
PTh	0.62	33.19	0.31	0.30	3×10^3	116.16[a]<br>108.12[b]	396	0.29	This work
PFu	0.65	3.15	0.36	0.30	8×10^2	10.66[a]<br>8.74[b]	47	0.23	This work
P(PyV)-H	0.55	54.2	1.58	0.18	$> 10^7$	120	485	0.25	[10]
P(gTDPP2FT)	0.64	15.34	1.75	0.15	5×10^6	54.8	156	0.35	[12]
BBL_{152}	0.15	11.1	0.38	0.15	4.4×10^5	25.9	589	4.4×10^{-2}	[13]
2DPP-OD-TEG	0.89	0.73	500	500	2×10^5	7	N.A.	N.A.	[14]
f-BTI2g-TVTCN	0.68	12.8	68	27	1×10^5	41.3	170	0.24	[15]
f-BTI2g-TVT	0.9	0.27	52	17	1×10^2	1.50	110	0.014	[15]
f-BTI2g-TVTF	0.75	22.6	53	4	1×10^5	90.2	222	0.41	[16]
f-BTI2g-SVSCN	0.70	48.3	7.3	3	$\sim 10^4$	150.9	347	0.36	[17]
f-BSeI2g-SVSCN	0.68	71.4	5.1	2	$\sim 10^4$	191.2	387	0.48	[17]
f-BTI2TEG-FT	0.53	4.6	272	35	1.3×10^3	15.20	443	0.034	[18]
CNg4T2-CNT2	0.75	6.75	102	4.2	10^4	27.01	309.03	0.087	[19]
FBDOPV-CNTVT	0.0075	1.58	21.7	34.8	N.A.	6.13	33.44	0.18	[20]

[a] Extracted from the slope of the $I_D^{0.5}$ vs. V_G linear fit in Figure S8. [b] Extracted from the slope of the g_m vs. WdL^{-1} $(V_G - V_{th})$ linear fit in Figure S10.

Table S1. Summary of n-type accumulation-mode OECT performance (continued).

**polymer**	V_{th} / V	$g_{m, norm}$ / S cm^{-1}	τ_{ON} / ms	τ_{OFF} / ms	I_{ON}/I_{OFF}	μC^* / F cm^{-1} V^{-1} s^{-1}	C^* / F cm^{-3}	μ / cm^2 V^{-1} s^{-1}	**REF**
p(C-V)	0.17	1.81	1.20	0.13	N.A.	14.89	126.75	0.117	[21]
p(C2F-V)	0.02	25.67	0.33	0.18	N.A.	107.56	117.96	0.912	[21]
p(C-T)	0.43	0.80	N.A.	N.A.	N.A.	6.7	97	0.069	[22]
p(N-T)	0.25	0.72	N.A.	N.A.	N.A.	4.3	73	0.059	[22]
p(C-2T)	0.44	0.14	N.A.	N.A.	N.A.	1.0	53	0.019	[22]
bgTNR-3OT	0.44	12.26	571	281	~ 10^4	54.3	116	0.47	[23]
bgTNR-3DT	0.51	13.30	634	175	~ 10^3	63.2	102	0.62	[23]
bgTNR-5OP	0.54	8.52	997	381	~ 10^3	43.0	89	0.48	[23]
p(C_6-T_2)	0.30	2.28	9.6	N.A.	N.A.	1.29	272	4.74×10^{-3}	[24]
gNDI-V	0.30	0.042	2.90	0.32	~ 10^5	2.31	144	1.4×10^{-2}	[25]
gNDI-T	0.20	0.11	0.87	0.18	~ 10^4	0.42	237	1.5×10^{-3}	[25]
P(DPP-TDP)	0.575	1.31	12.5	0.6	N.A.	7.62	68.58	0.11	[26]
n-PT3	0.56	40.4	23.3	2.3	1.7×10^6	91.8	103	0.89	[27]
o-CNgTVT-2FT	0.79	17.4	2.86	0.33	~ 10^7	100.0	193.3	0.51	[28]

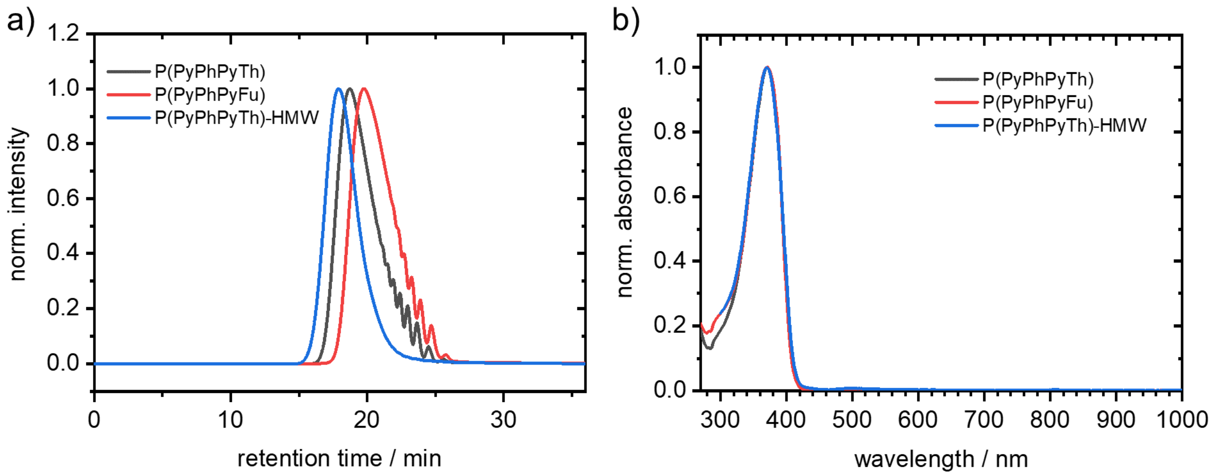

Figure S1. a) SEC elution curves (THF) of the non-quaternised polymers. b) UV-vis spectra of the non-quaternised polymers at 25 °C in THF.

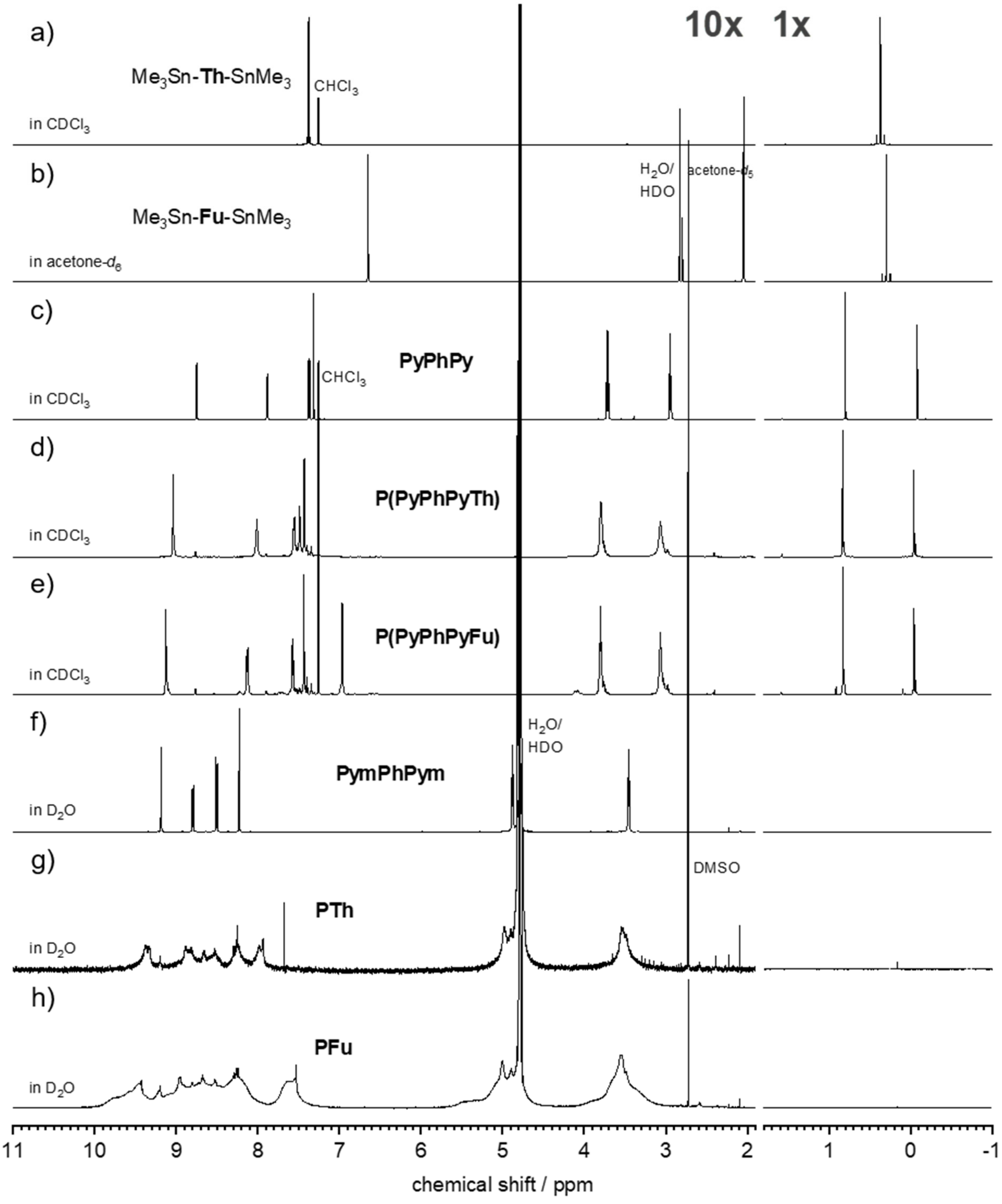

Figure S2. Full-range ^{1}H NMR spectra of a) – c) monomers, d) – e) non-quaternised polymers, f) quaternised monomer, and g) – h) quaternised polymers. The signals in the left panel are magnified compared to the right panel by a factor of 10.

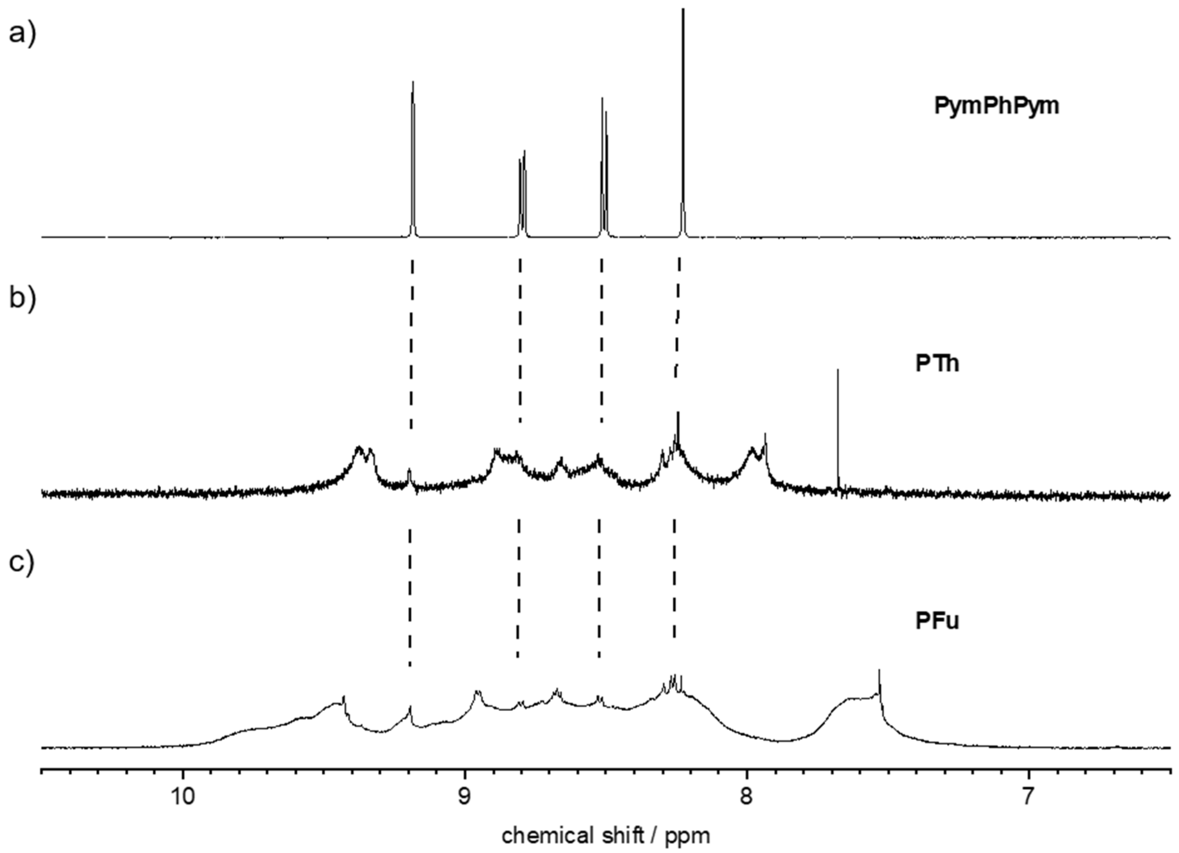

Figure S3. ^{1}H NMR spectra (aromatic region) of a) PymPhPym, b) PTh, and c) PFu. The dashed lines are guides to the eye, indicating the presence of sharper end-group signals in the broad polymer spectra.

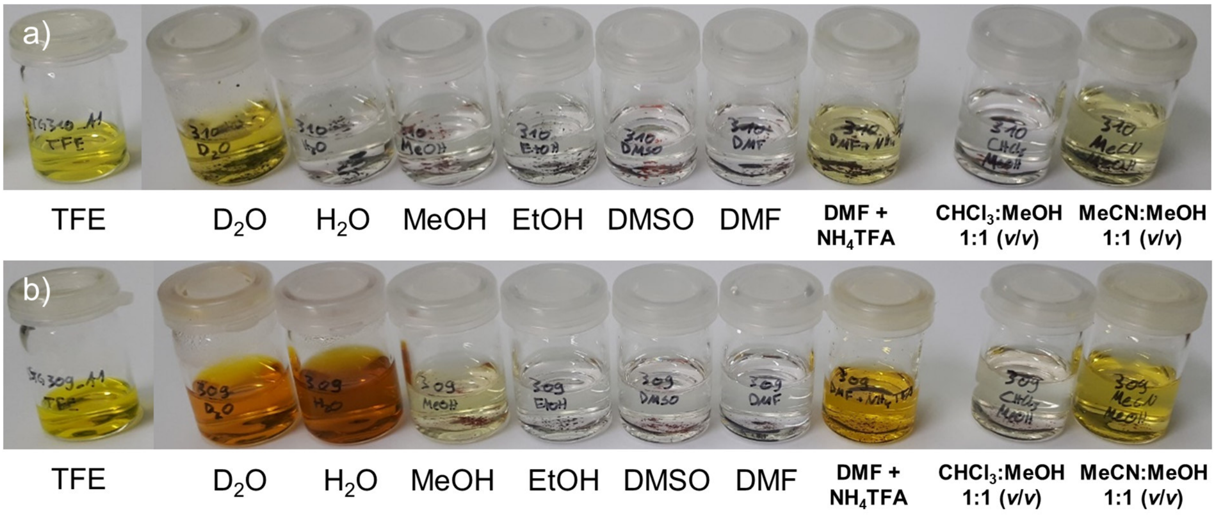

Figure S4. Qualitative solubility test of a) PTh and b) PFu in different solvents, electrolyte solutions, and solvent mixtures.

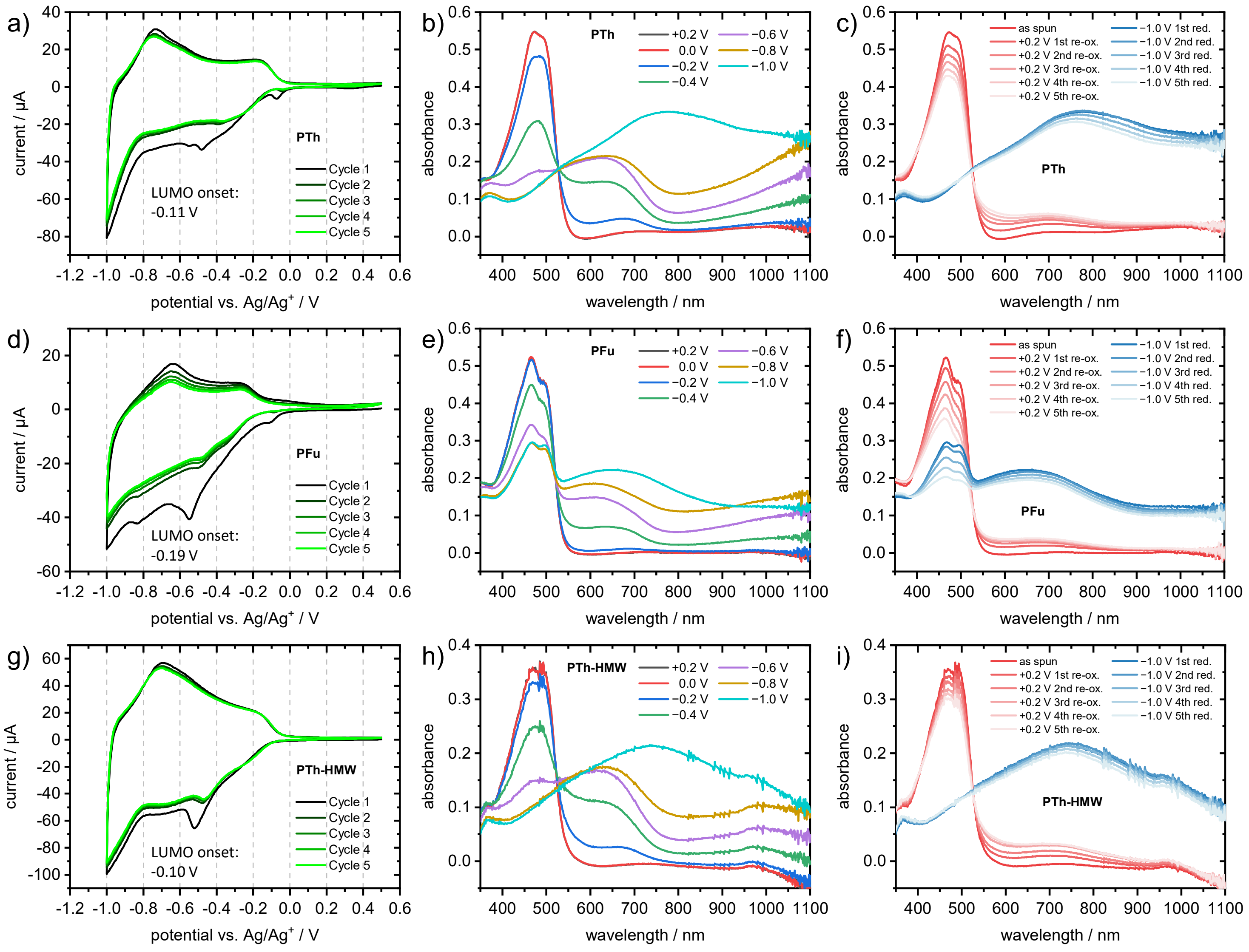

Figure S5. Spectro-electrochemistry of the polymers. a) – c) PTh, d) – f) PFu, and g) – i) PTh-HMW.

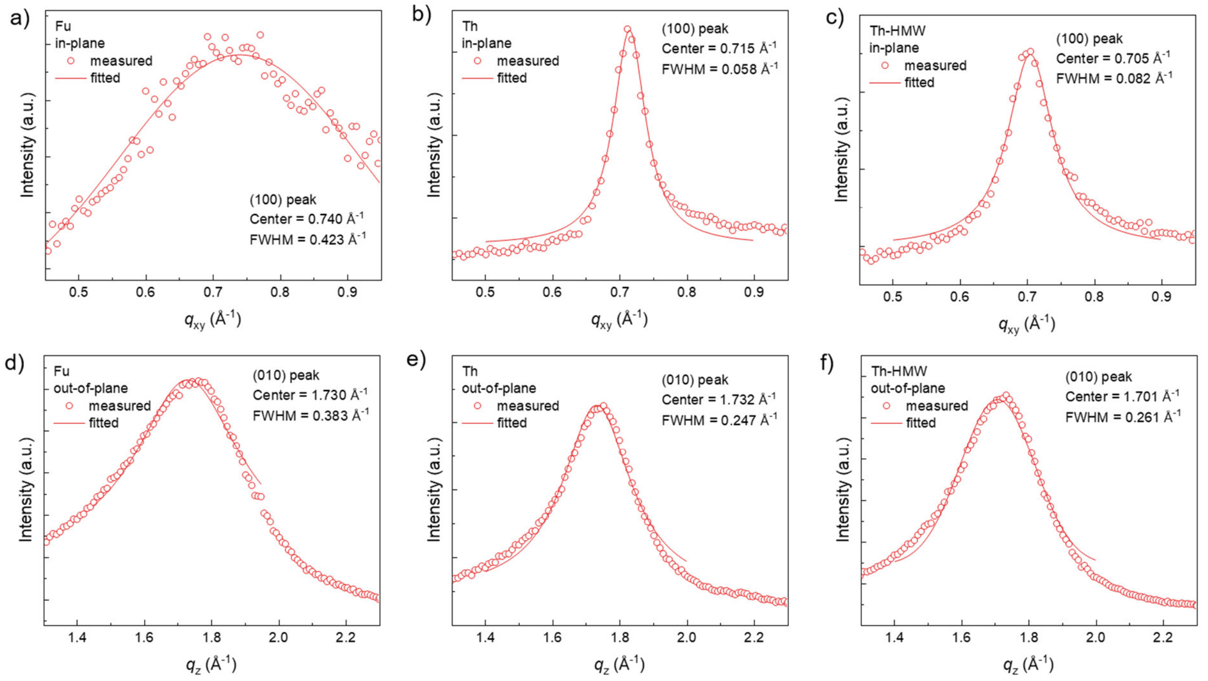

Figure S6. GIWAXS diffraction peak analysis. (100) Lamellar stacking peak of a) PFu, b) PTh, and c) PTh-HMW. (010) π–π stacking peak of d) PFu, e) PTh, and f) PTh-HMW.

Table S2. Summary of GIWAXS results.

	π-π stacking				Lamellar stacking			
polymer	q_z (010) (Å^{-1})	Distance (Å)	Coherence length (Å)	Paracrystalline disorder	q_{xy} (100) (Å^{-1})	Distance (Å)	Coherence length (Å)	Paracrystalline disorder
PFu	1.730	3.632	17.764	0.188	0.740	8.491	13.368	0.303
PTh	1.732	3.628	22.894	0.151	0.715	8.788	97.498	0.114
PTh-HMW	1.701	3.694	21.666	0.156	0.705	8.912	68.962	0.136

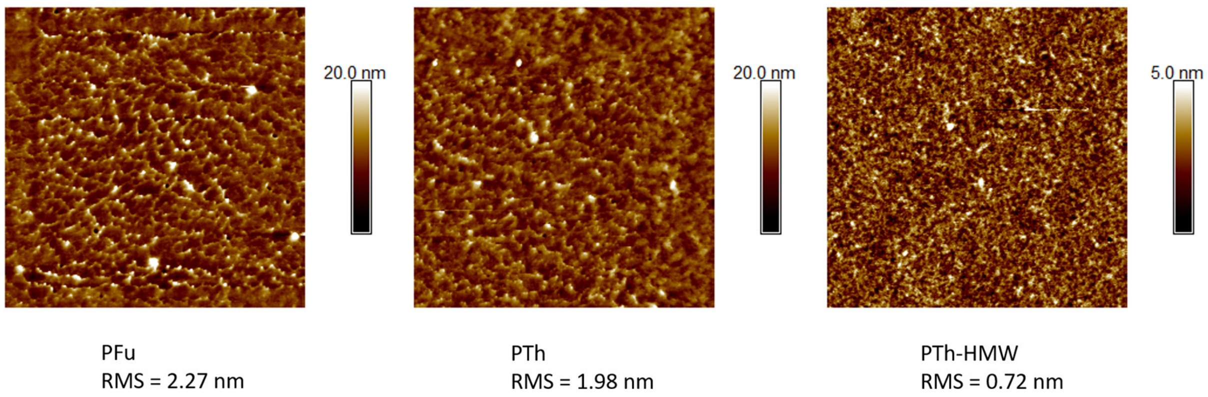

Figure S7. AFM height images of spin-coated thin films of the quaternised polymers. The size of the images is 2 µm x 2 µm.

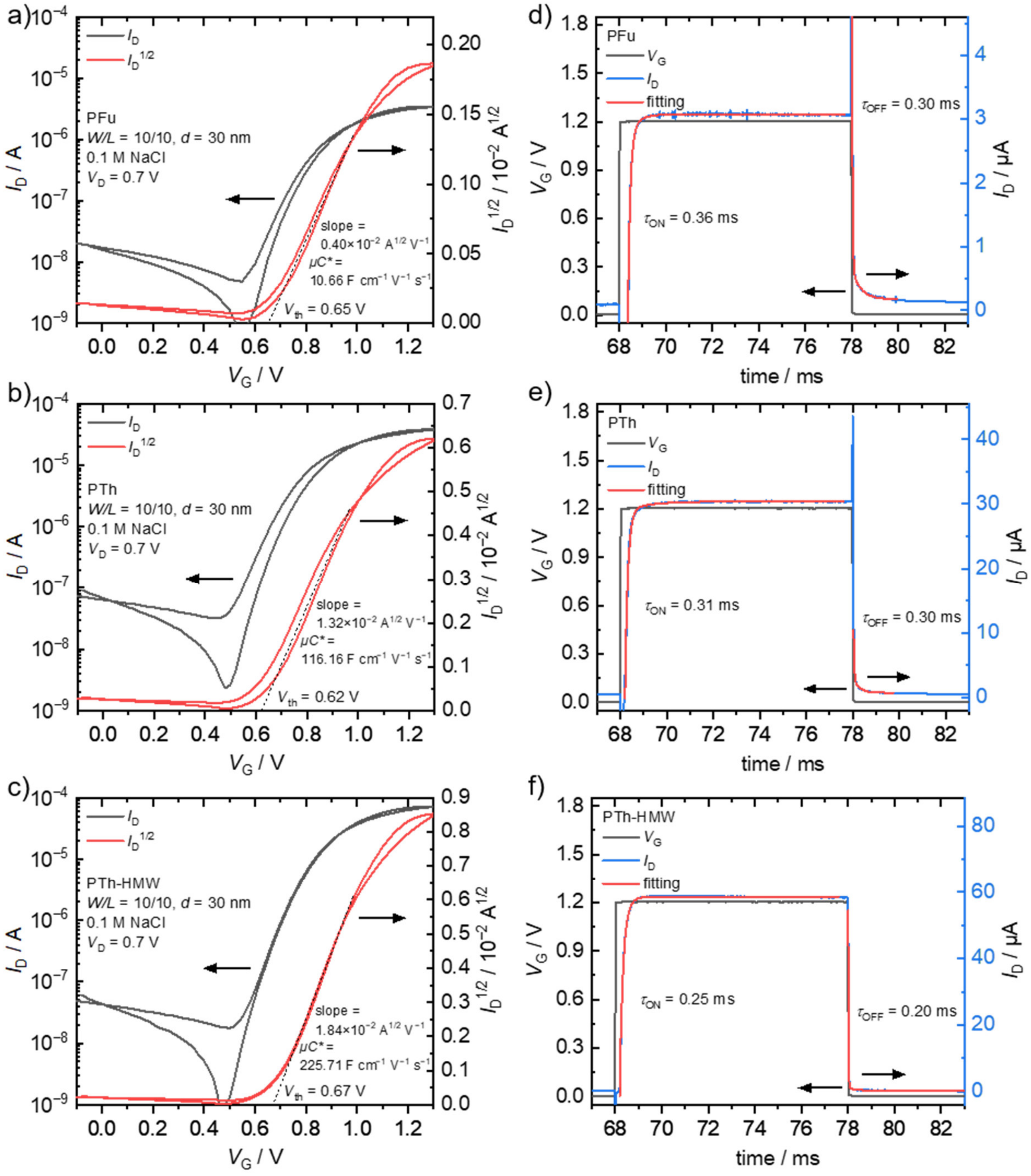

Figure S8. a) – c) Transfer curves and d) – f) transient response of OECTs based on PFu (a,d), PTh (b,e), and PTh-HMW (c,f). The transfer curves display the drain current (I_D) and its square root ($I_D^{1/2}$). The fitted threshold voltage (V_{th}) and μC^* were also reported. For the transient response, gate voltage (V_G) pulses were applied from 0 V to 1.2 V, and the corresponding mean lifetimes τ_{on} and τ_{off} were reported. All OECTs had the same channel geometry (W = 10 µm, L = 10 µm, and d = 30 nm).

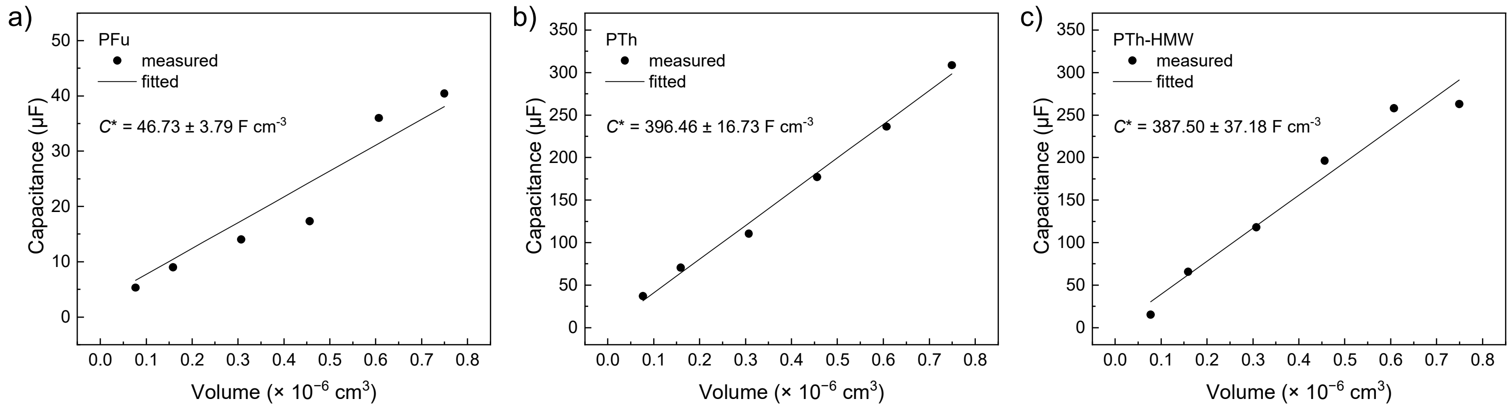

Figure S9. Volume-dependent capacitance of patterned thin films of a) PFu, b) PTh, and c) PTh-HMW. The capacitances were determined from the EIS spectra, and the volumetric capacitances *C** are calculated from linear fitting of the volume-dependent capacitances.

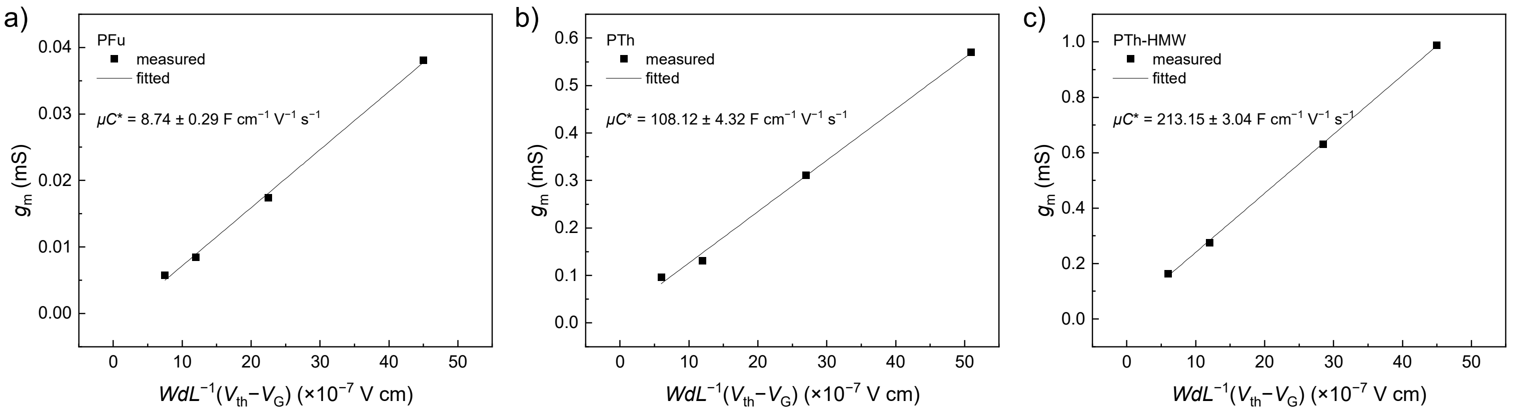

Figure S10. The g_m value of pristine a) PFu, b) PTh, and c) PTh-HMW-based OECTs with varying channel geometry.

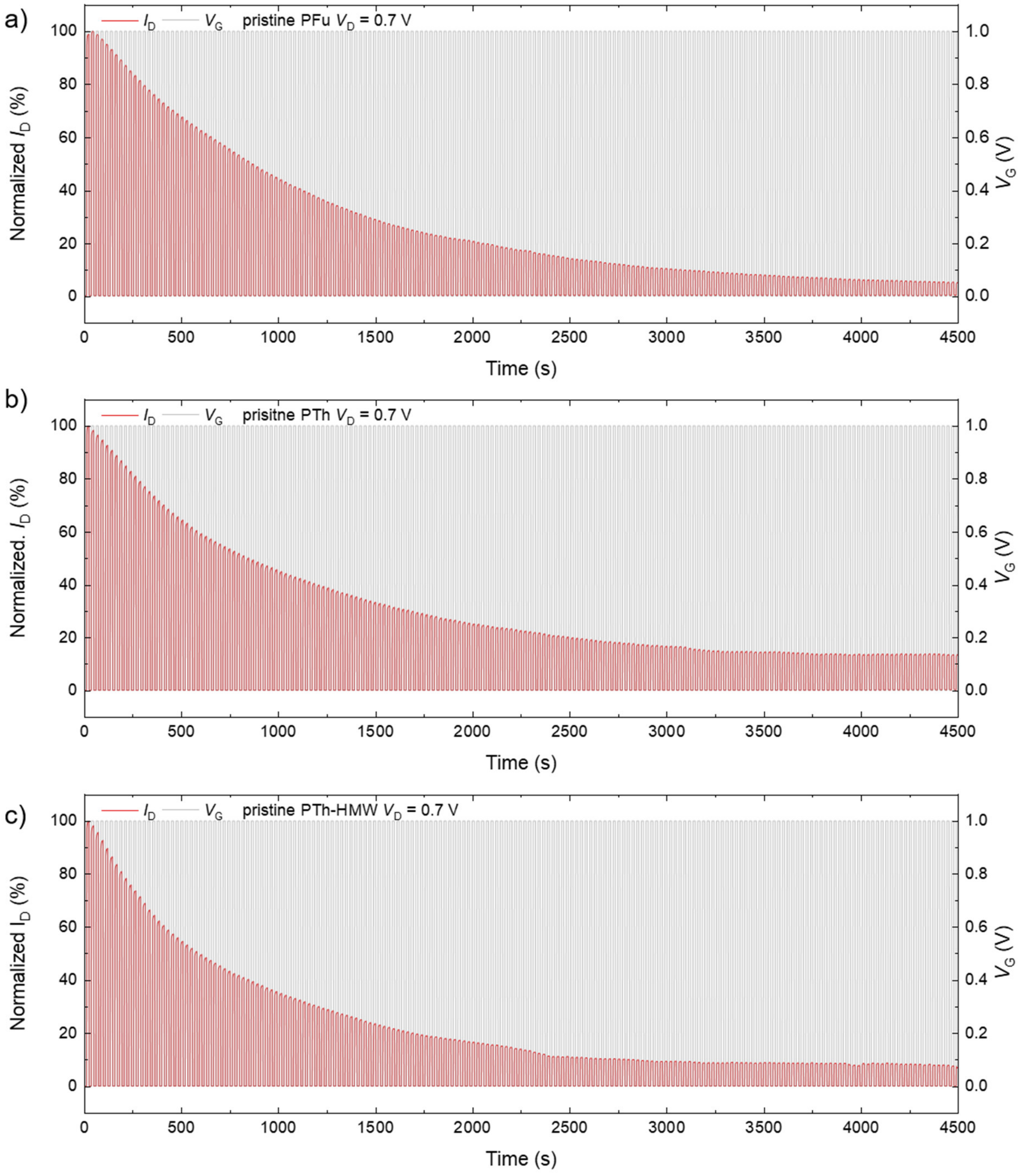

Figure S11. Operational stability of pristine a) PFu, b) PTh, and c) PTh-HMW-based OECTs. Gate voltage (V_G) pulses were applied from 0 to 1.0 V. We expect that the solubility of the polymers in water and electrolyte solutions (cf. Figure S4) affect the stability of the devices.

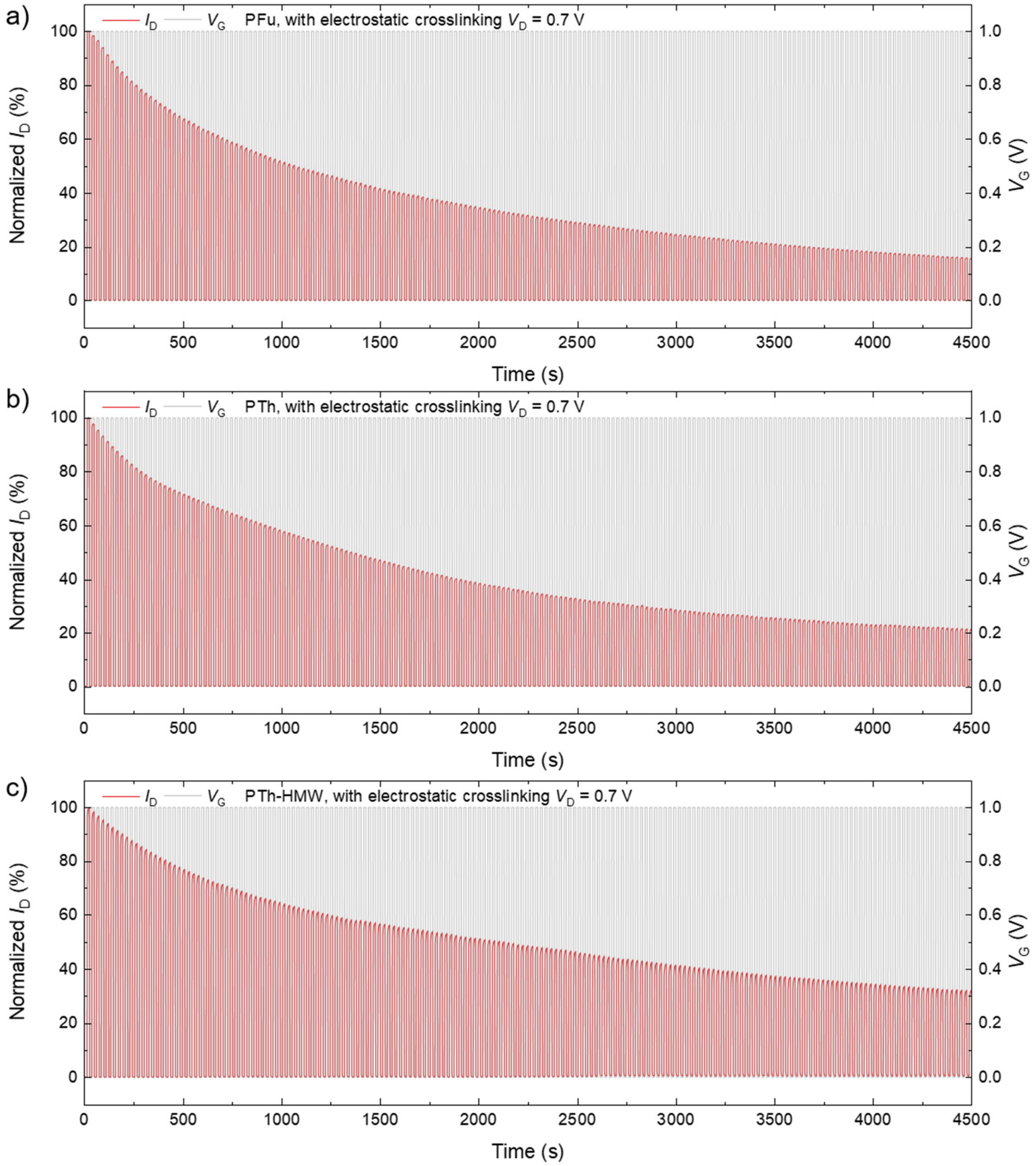

Figure S12. Operational stability of electrostatically crosslinked a) PFu, b) PTh, and c) PTh-HMW-based OECTs. Gate voltage (V_G) pulses were applied from 0 to 1.0 V.

Additional NMR Spectra

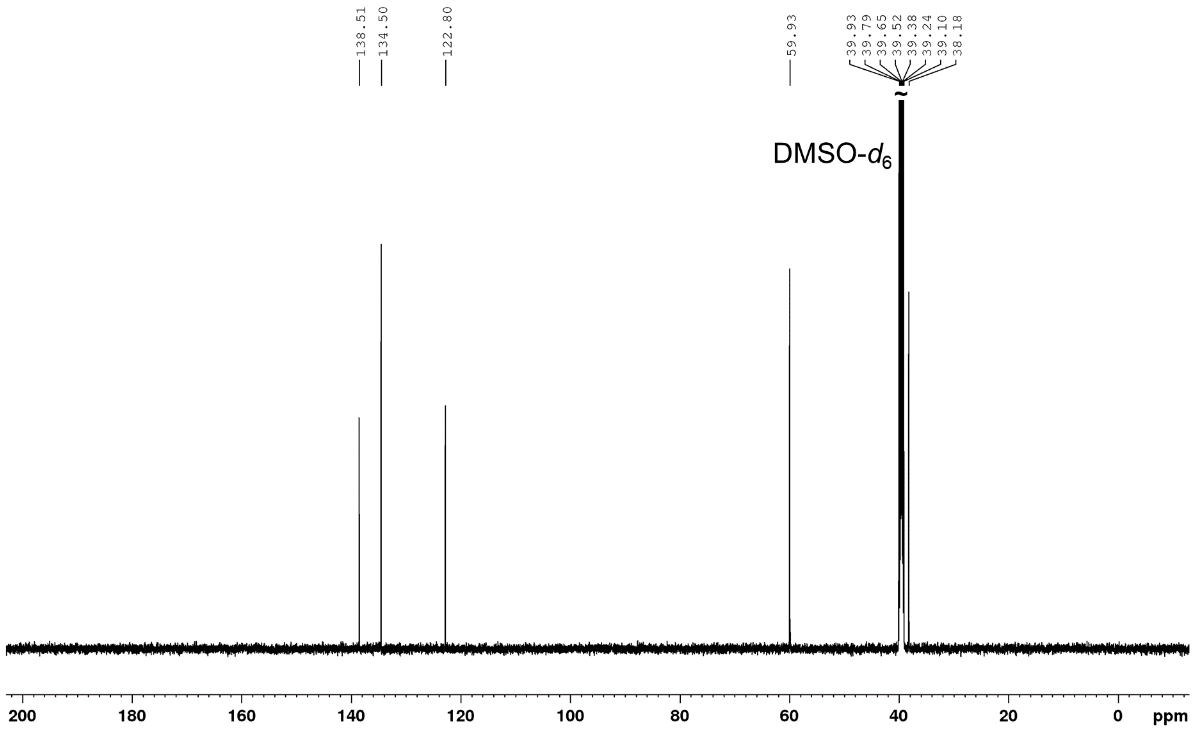

Figure S13. $^{13}C\{^1H\}$ NMR spectrum (DMSO-d_6, 151 MHz, 25 °C) of 3.

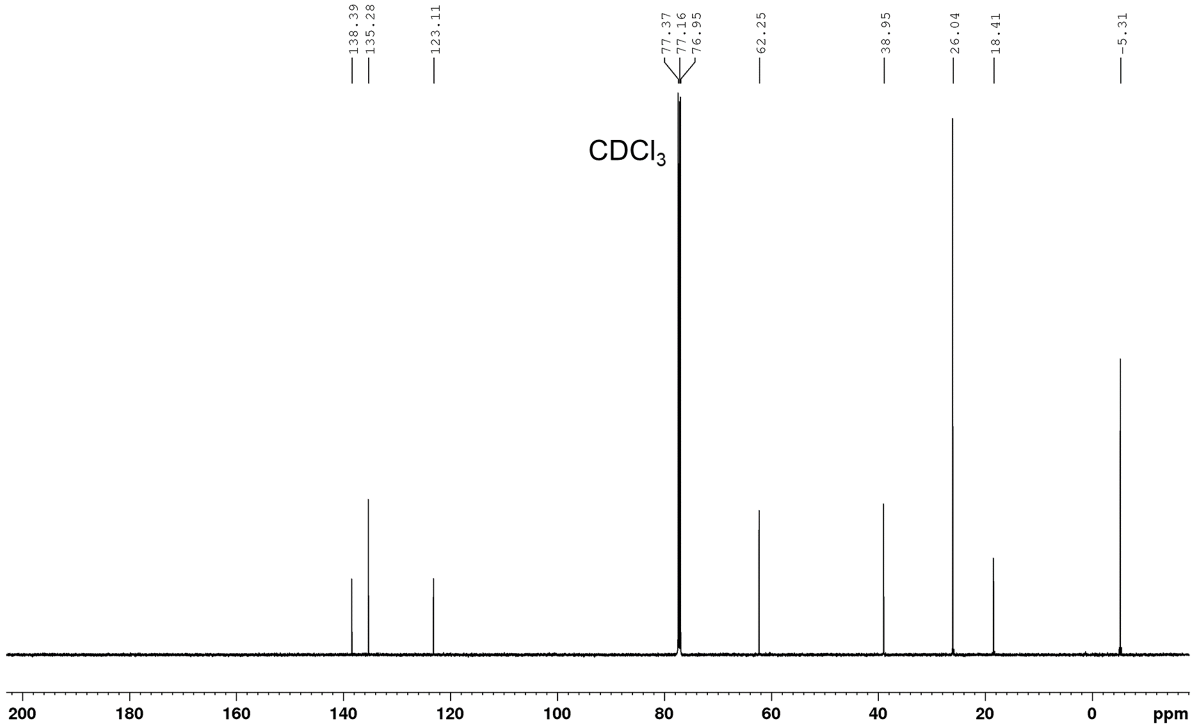

Figure S14. $^{13}C\{^1H\}$ NMR spectrum ($CDCl_3$, 151 MHz, 25 °C) of 4.

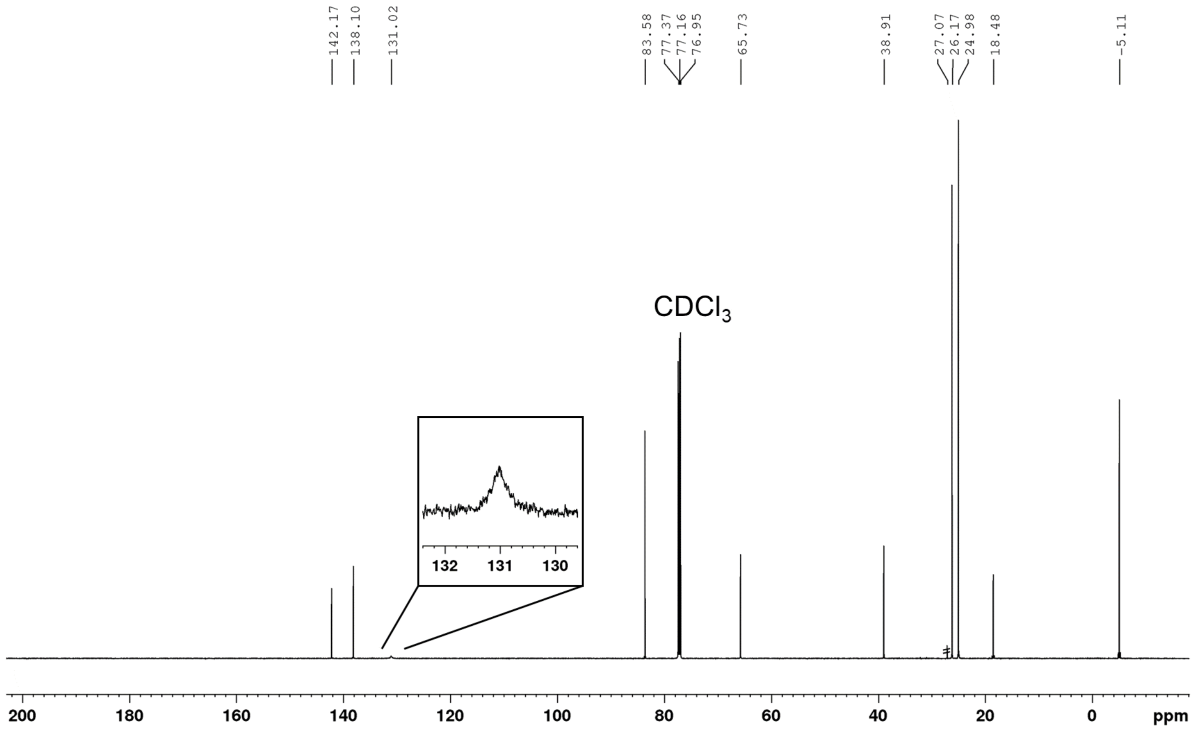

Figure S15. $^{13}C\{^1H\}$ NMR spectrum ($CDCl_3$, 151 MHz, 25 °C) of 5.

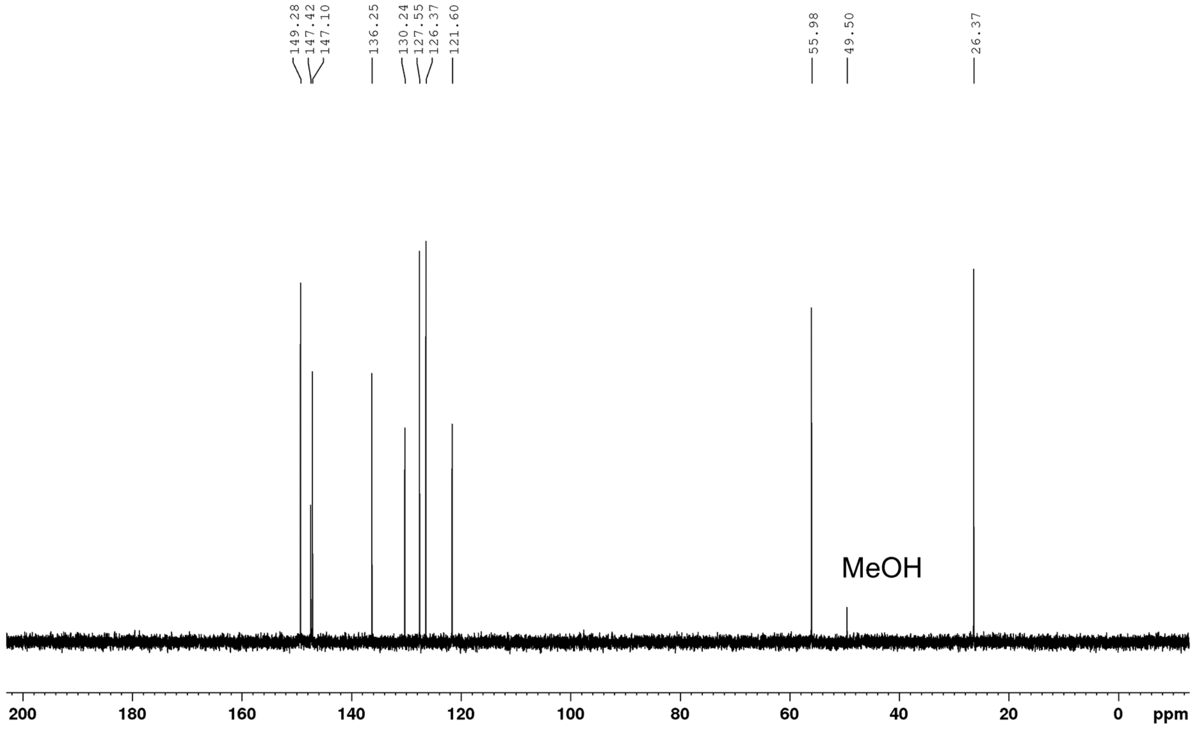

Figure S16. $^{13}C\{^1H\}$ NMR spectrum (D_2O, 151 MHz, 25 °C) of PymPhPym.

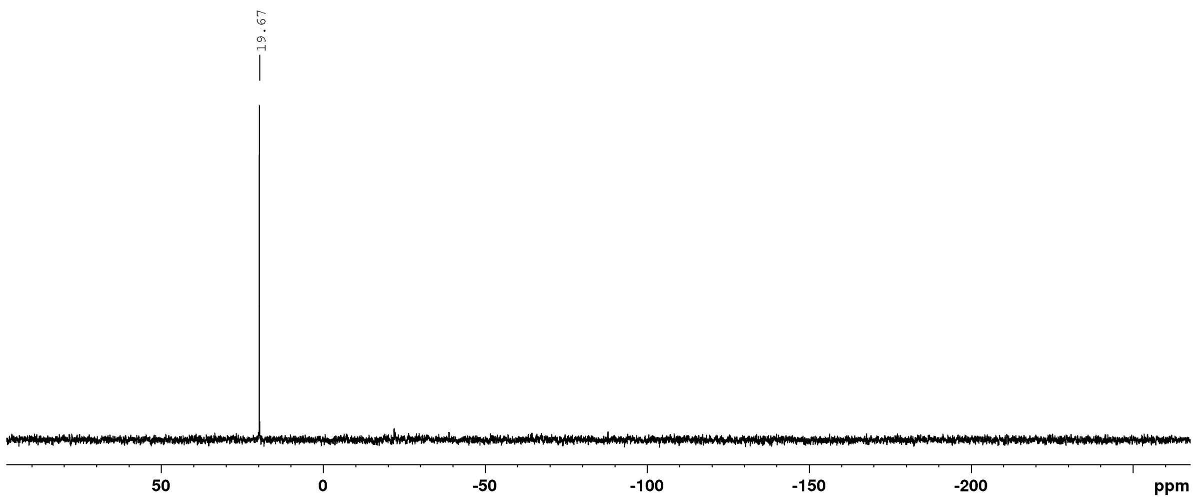

Figure S17. $^{29}Si\{^1H\}$ NMR spectrum ($CDCl_3$, 119 MHz, 25 °C) of 4.

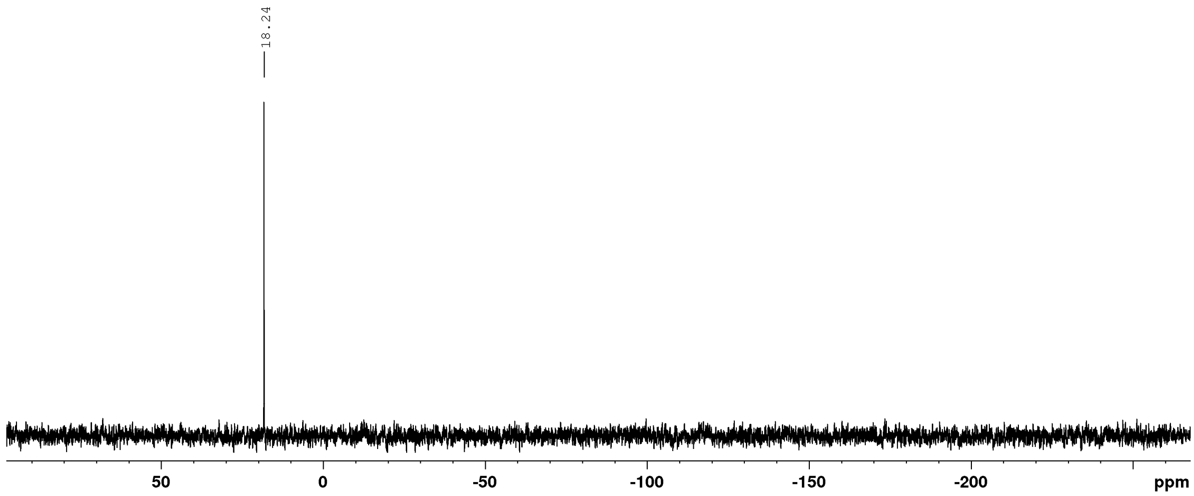

Figure S18. $^{29}Si\{^1H\}$ NMR spectrum ($CDCl_3$, 119 MHz, 25 °C) of 5.

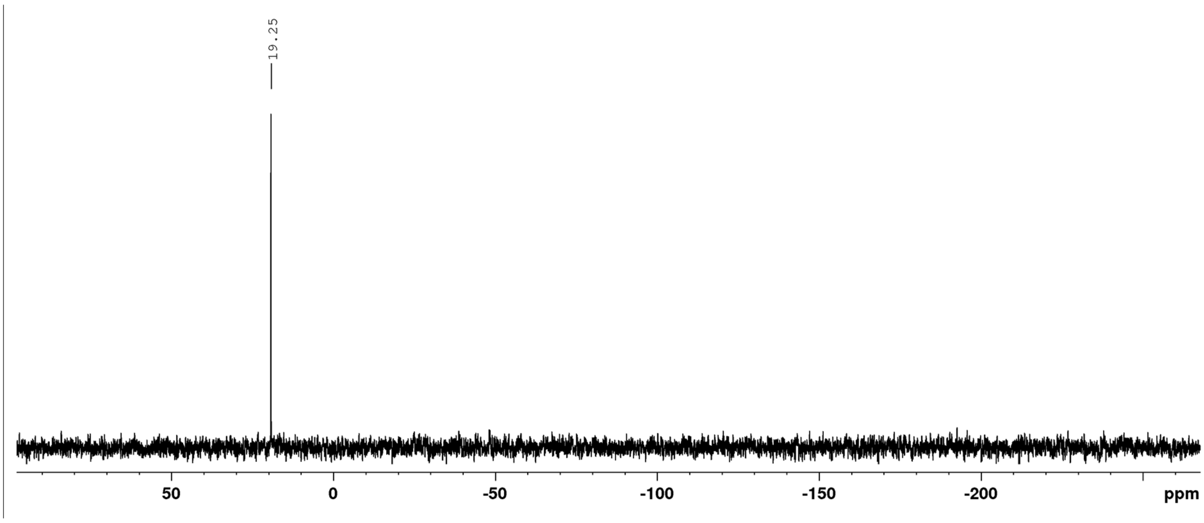

Figure S19. $^{29}Si\{^1H\}$ NMR spectrum ($CDCl_3$, 119 MHz, 25 °C) of PyPhPy.

References

(1) Fan, S.; Wang, Z.; Li, C.; Liang, Y.; Chen, T.; Jin, L. Y. Self-Assembly of Coil-Rod-Coil Triblock Copolymers Depending on Lateral Methyl Groups at the Interface of Rod and Coil Segments. *Macromol. Res.* **2015**, *23* (10), 909–915. https://doi.org/10.1007/s13233-015-3122-1.

(2) Bonifacio, M. C.; Robertson, C. R.; Jung, J.-Y.; King, B. T. Polycyclic Aromatic Hydrocarbons by Ring-Closing Metathesis. *J. Org. Chem.* **2005**, *70* (21), 8522–8526. https://doi.org/10.1021/jo051418o.

(3) Izuhara, D.; Swager, T. M. Poly(Pyridinium Phenylene)s: Water-Soluble N-Type Polymers. *J. Am. Chem. Soc.* **2009**, *131* (49), 17724–17725. https://doi.org/10.1021/ja906513u.

(4) Peters, G. M.; Grover, G.; Maust, R. L.; Colwell, C. E.; Bates, H.; Edgell, W. A.; Jasti, R.; Kertesz, M.; Tovar, J. D. Linear and Radial Conjugation in Extended π-Electron Systems. *J. Am. Chem. Soc.* **2020**, *142* (5), 2293–2300. https://doi.org/10.1021/jacs.9b10785.

(5) Seitz, D. E.; Lee, S.-H.; Hanson, R. N.; Bottaro, J. C. Synthesis and Reactivity of the 2,5-Bis(Trimethylstannyl) Derivatives of Thiophene and Furan. *Synth. Commun.* **1983**, *13* (2), 121–128. https://doi.org/10.1080/00397918308061969.

(6) Cardona, C. M.; Li, W.; Kaifer, A. E.; Stockdale, D.; Bazan, G. C. Electrochemical Considerations for Determining Absolute Frontier Orbital Energy Levels of Conjugated Polymers for Solar Cell Applications. *Adv. Mater.* **2011**, *23* (20), 2367–2371. https://doi.org/10.1002/adma.201004554.

(7) Wu, H.-Y.; Huang, J.-D.; Jeong, S. Y.; Liu, T.; Wu, Z.; Pol, T. van der; Wang, Q.; Stoeckel, M.-A.; Li, Q.; Fahlman, M.; Tu, D.; Woo, H. Y.; Yang, C.-Y.; Fabiano, S. Stable Organic Electrochemical Neurons Based on P-Type and n-Type Ladder Polymers. *Mater. Horiz.* **2023**, *10* (10), 4213–4223. https://doi.org/10.1039/D3MH00858D.

(8) Peng, Z.; Ye, L.; Ade, H. Understanding, Quantifying, and Controlling the Molecular Ordering of Semiconducting Polymers: From Novices to Experts and Amorphous to Perfect Crystals. *Mater. Horiz.* **2022**, *9* (2), 577–606. https://doi.org/10.1039/D0MH00837K.

(9) Hwang, S.; Potscavage, W. J.; Yang, Y. S.; Park, I. S.; Matsushima, T.; Adachi, C. Solution-Processed Organic Thermoelectric Materials Exhibiting Doping-Concentration-Dependent Polarity. *Phys. Chem. Chem. Phys.* **2016**, *18* (42), 29199–29207. https://doi.org/10.1039/C6CP04572C.

(10) Li, P.; Sun, W.; Li, J.; Chen, J.-P.; Wang, X.; Mei, Z.; Jin, G.; Lei, Y.; Xin, R.; Yang, M.; Xu, J.; Pan, X.; Song, C.; Deng, X.-Y.; Lei, X.; Liu, K.; Wang, X.; Zheng, Y.; Zhu, J.; Lv, S.; Zhang, Z.; Dai, X.; Lei, T. N-Type Semiconducting Hydrogel. *Science* **2024**, *384* (6695), 557–563. https://doi.org/10.1126/science.adj4397.

(11) Li, J.-L.; Deng, X.-Y.; Chen, J.; Fu, P.-X.; Tian, S.-Y.; Wang, Y.; Gu, X.; Lei, T. Cationic Conjugated Polymers with Enhanced Doped-State Planarity for n-Type Organic Thermoelectrics. *CCS Chem.* **2025**, *7* (5), 1449–1458. https://doi.org/10.31635/ccschem.024.202404274.

(12) Li, P.; Shi, J.; Lei, Y.; Huang, Z.; Lei, T. Switching P-Type to High-Performance n-Type Organic Electrochemical Transistors via Doped State Engineering. *Nat. Commun.* **2022**, *13* (1), 5970. https://doi.org/10.1038/s41467-022-33553-w.

(13) Wu, H.-Y.; Yang, C.-Y.; Li, Q.; Kolhe, N. B.; Strakosas, X.; Stoeckel, M.-A.; Wu, Z.; Jin, W.; Savvakis, M.; Kroon, R.; Tu, D.; Woo, H. Y.; Berggren, M.; Jenekhe, S. A.; Fabiano, S. Influence of Molecular Weight on the Organic Electrochemical Transistor Performance of Ladder-Type Conjugated Polymers. *Adv. Mater.* **2022**, *34* (4), 2106235. https://doi.org/10.1002/adma.202106235.

(14) Samuel, J. J.; Garudapalli, A.; Mohapatra, A. A.; Gangadharappa, C.; Patil, S.; Aetukuri, N. P. B. Single-Component CMOS-Like Logic Using Diketopyrrolopyrrole-Based

Ambipolar Organic Electrochemical Transistors. *Adv. Funct. Mater.* **2021**, *31* (45), 2102903. https://doi.org/10.1002/adfm.202102903.

(15) Feng, K.; Shan, W.; Wang, J.; Lee, J.-W.; Yang, W.; Wu, W.; Wang, Y.; Kim, B. J.; Guo, X.; Guo, H. Cyano-Functionalized n-Type Polymer with High Electron Mobility for High-Performance Organic Electrochemical Transistors. *Adv. Mater.* **2022**, *34* (24), 2201340. https://doi.org/10.1002/adma.202201340.

(16) Yang, W.; Feng, K.; Ma, S.; Liu, B.; Wang, Y.; Ding, R.; Jeong, S. Y.; Woo, H. Y.; Chan, P. K. L.; Guo, X. High-Performance n-Type Polymeric Mixed Ionic-Electronic Conductors: The Impacts of Halogen Functionalization. *Adv. Mater.* **2024**, *36* (4), 2305416. https://doi.org/10.1002/adma.202305416.

(17) Wu, W.; Feng, K.; Wang, Y.; Wang, J.; Huang, E.; Li, Y.; Jeong, S. Y.; Woo, H. Y.; Yang, K.; Guo, X. Selenophene Substitution Enabled High-Performance n-Type Polymeric Mixed Ionic-Electronic Conductors for Organic Electrochemical Transistors and Glucose Sensors. *Adv. Mater.* **2024**, *36* (1), 2310503. https://doi.org/10.1002/adma.202310503.

(18) Feng, K.; Shan, W.; Ma, S.; Wu, Z.; Chen, J.; Guo, H.; Liu, B.; Wang, J.; Li, B.; Woo, H. Y.; Fabiano, S.; Huang, W.; Guo, X. Fused Bithiophene Imide Dimer-Based n-Type Polymers for High-Performance Organic Electrochemical Transistors. *Angew. Chem. Int. Ed.* **2021**, *60* (45), 24198–24205. https://doi.org/10.1002/anie.202109281.

(19) Ma, S.; Wang, J.; Wu, W.; Wu, Z.; Zhang, H.; Chen, R.; Liu, B.; Feng, K.; Woo, H. Y.; Guo, X. Sequential Cyanation of Polythiophenes: Tuning Charge Carrier Polarity in Organic Electrochemical Transistors. *Adv. Electron. Mater.* **2023**, *9* (11), 2300207. https://doi.org/10.1002/aelm.202300207.

(20) Ding, R.; Zhang, X.; Yan, R.; Peng, M.; Su, S.; Jeong, S. Y.; Woo, H. Y.; Guo, X.; Feng, K.; Guo, Z.-H. Ultra-Low Threshold Voltage in N-Type Organic Electrochemical Transistors Enabled by Organic Mixed Ionic-Electronic Conductors with Dual Electron-Withdrawing Substitutions. *Adv. Funct. Mater.* **2025**, *35* (2), 2412181. https://doi.org/10.1002/adfm.202412181.

(21) Wang, Y.; Koklu, A.; Zhong, Y.; Chang, T.; Guo, K.; Zhao, C.; Castillo, T. C. H.; Bu, Z.; Xiao, C.; Yue, W.; Ma, W.; Inal, S. Acceptor Functionalization via Green Chemistry Enables High-Performance n-Type Organic Electrochemical Transistors for Biosensing, Memory Applications. *Adv. Funct. Mater.* **2024**, *34* (15), 2304103. https://doi.org/10.1002/adfm.202304103.

(22) Wang, Y.; Zeglio, E.; Wang, L.; Cong, S.; Zhu, G.; Liao, H.; Duan, J.; Zhou, Y.; Li, Z.; Mawad, D.; Herland, A.; Yue, W.; McCulloch, I. Green Synthesis of Lactone-Based Conjugated Polymers for n-Type Organic Electrochemical Transistors. *Adv. Funct. Mater.* **2022**, *32* (16), 2111439. https://doi.org/10.1002/adfm.202111439.

(23) Duan, J.; Xiao, M.; Zhu, G.; Chen, J.; Hou, H.; Gámez-Valenzuela, S.; Zelewski, S. J.; Dai, L.; Tao, X.; Ran, C.; Jay, N.; Lin, Y.; Guo, X.; Yue, W. Molecular Ordering Manipulation in Fused Oligomeric Mixed Conductors for High-Performance n-Type Organic Electrochemical Transistors. *ACS Nano* **2024**, *18* (41), 28070–28080. https://doi.org/10.1021/acsnano.4c07219.

(24) Ohayon, D.; Savva, A.; Du, W.; Paulsen, B. D.; Uguz, I.; Ashraf, R. S.; Rivnay, J.; McCulloch, I.; Inal, S. Influence of Side Chains on the N-Type Organic Electrochemical Transistor Performance. *ACS Appl. Mater. Interfaces* **2021**, *13* (3), 4253–4266. https://doi.org/10.1021/acsami.0c18599.

(25) Chen, J.; Cong, S.; Wang, L.; Wang, Y.; Lan, L.; Chen, C.; Zhou, Y.; Li, Z.; McCulloch, I.; Yue, W. Backbone Coplanarity Manipulation via Hydrogen Bonding to Boost the N-Type Performance of Polymeric Mixed Conductors Operating in Aqueous Electrolyte. *Mater. Horiz.* **2023**, *10* (2), 607–618. https://doi.org/10.1039/D2MH01100J.

(26) Erhardt, A.; Hochgesang, A.; McNeill, C. R.; Thelakkat, M. A Competitive N-Type OECT Material via Copolymerization of Electron Deficient Building Blocks. *Adv. Electron. Mater.* **2023**, *9* (7), 2300026. https://doi.org/10.1002/aelm.202300026.
(27) Kuang, Y.; Yao, T.; Deng, S.; Dong, J.; Ye, G.; Zhang, L.; Shao, S.; Zhu, Z.; Liu, J.; Liu, J. Matching P- and N-Type Organic Electrochemical Transistor Performance Enables a Record High-Gain Complementary Inverter. *Adv. Mater.* **2025**, *37* (7), 2417691. https://doi.org/10.1002/adma.202417691.
(28) Zhang, C.; Zheng, Y.; Li, Y.; Xue, Z.; Zhu, X.; Chen, J.; Ma, J.; Zhang, Z.; Zhong, H.; Yue, W.; Lei, T.; Fei, Z. Polythiophenes for High-Performance N-Type Organic Electrochemical Transistors. *Adv. Funct. Mater.* **2025**, *35* (23), 2419706. https://doi.org/10.1002/adfm.202419706.
